# Glucose-*ABL1*-TOR Signaling Modulates Cell Cycle Tuning to Control Terminal Appressorial Cell Differentiation

**DOI:** 10.1371/journal.pgen.1006557

**Published:** 2017-01-10

**Authors:** Margarita Marroquin-Guzman, Guangchao Sun, Richard A. Wilson

**Affiliations:** Department of Plant Pathology, University of Nebraska-Lincoln, Lincoln, Nebraska, United States of America; The University of North Carolina at Chapel Hill, UNITED STATES

## Abstract

The conserved target of rapamycin (TOR) pathway integrates growth and development with available nutrients, but how cellular glucose controls TOR function and signaling is poorly understood. Here, we provide functional evidence from the devastating rice blast fungus *Magnaporthe oryzae* that glucose can mediate TOR activity via the product of a novel carbon-responsive gene, *ABL1*, in order to tune cell cycle progression during infection-related development. Under nutrient-free conditions, wild type (WT) *M*. *oryzae* strains form terminal plant-infecting cells (appressoria) at the tips of germ tubes emerging from three-celled spores (conidia). WT appressorial development is accompanied by one round of mitosis followed by autophagic cell death of the conidium. In contrast, Δ*abl1* mutant strains undergo multiple rounds of accelerated mitosis in elongated germ tubes, produce few appressoria, and are abolished for autophagy. Treating WT spores with glucose or 2-deoxyglucose phenocopied Δ*abl1*. Inactivating TOR in Δ*abl1* mutants or glucose-treated WT strains restored appressorium formation by promoting mitotic arrest at G1/G0 via an appressorium- and autophagy-inducing cell cycle delay at G2/M. Collectively, this work uncovers a novel glucose-*ABL1*-TOR signaling axis and shows it engages two metabolic checkpoints in order to modulate cell cycle tuning and mediate terminal appressorial cell differentiation. We thus provide new molecular insights into TOR regulation and cell development in response to glucose.

## Author Summary

TOR kinase coordinates cell development with nutrient availability. Due to its roles in many debilitating diseases, it is important that all aspects of TOR regulation are understood. Nonetheless, how cellular glucose controls TOR function and downstream activities is largely unknown. Here, we discovered and characterized a negative-acting TOR regulator, encoded by *ABL1*, necessary for infection-related development of the rice pathogen *Magnaporthe oryzae*. During spore germination, in the absence of glucose, the Abl1 protein inactivates TOR at two cell cycle checkpoints. This results in one round of prolonged mitosis that promotes autophagy and induces differentiation of the specialized rice-infecting appressorial cell. In contrast, glucose or glucose-6-phosphate (but not downstream metabolites) inactivates the Abl1 protein. This in turn activates TOR signaling to promote multiple, brisk rounds of mitosis that abolishes the cell cycle checkpoint delays necessary for inducing appressoria. We thus reveal a novel glucose-*ABL1*-TOR signaling axis controlling appressorial differentiation by modulating cell cycle progression in response to glucose. Together, our results are significant because they provide molecular insights on three poorly elucidated aspects of cell growth and development in any system: how glucose can regulate TOR, how glucose can regulate the cell cycle, and how TOR receives inhibitory signals.

## Introduction

The conserved TOR signaling pathway controls cell growth and proliferation across taxa and disease states by regulating metabolic processes in response to available nutrients and energy [1–4]. Amino acids signal to TOR directly via a class of small GTPases that, in mammalian cells, recruit TORC1 to the lysosome for activation under nitrogen/ amino acid sufficiency [5, 6]. During energy stress, the AMPK/Snf complex negatively regulates TOR pathway branches via phosphorylation of the downstream TOR component Raptor/Kog1 [7], although this is indirect in yeast [4]. In contrast to amino acids and energy, little is known about the molecular mechanisms underlying cellular glucose control of TOR signaling [3,8]. In yeast [1] and mammals [9], TOR kinase forms two complexes, TORC1 and TORC2, with different roles in cell growth. TORC1 is rapamycin sensitive and exerts temporal control on cell growth by regulating ribosome biogenesis in addition to protein, lipid and nucleotide biosynthesis. TORC1 inhibits autophagy when active. TORC2 is rapamycin insensitive and governs actin cytoskeleton organization during the cell cycle [1,10,11]. Loss of TORC1 components, or rapamycin treatment, results in cell cycle arrest at G1/G0 [1,12,13] and autophagy induction [1,14]. Temperature-sensitive yeast Kog1 mutants have shown that under nutrient-poor or rapamycin treatment conditions, TORC1 first delays the cell cycle at G2/M and induces autophagy before progressing mitosis to G1/G0 arrest [11, 14].

The fungus *Magnaporthe oryzae* causes blast, the most devastating disease of cultivated rice (*Oryza sativa*) [15–17]. Fungal spores attached to rice leaf surfaces elaborate specialized appressorial cells at germ tube tips that function to penetrate into underlying tissues where *M*. *oryzae* grows as parasitic invasive hyphae (IH). Recently, we have demonstrated status-dependent roles for TOR signaling that are critical for disease progression by *M*. *oryzae*: inactive or downregulated TOR signaling (TOR_off_) permits appressorial development on the rice leaf surface; activated TOR (TOR_on_) facilitates proliferation in rice cells [18,19]. Considering TOR kinase is likely to be intrinsically active [20], identifying factors that maintain TOR_off_ during appressorium formation is thus an essential but enigmatic component of our understanding of the rice infection process.

Two opposing signaling pathways, cAMP/PKA and TOR, regulate appressorial development. cAMP/PKA signaling is a positive-acting determinant of appressorial development [21], whereas TOR signaling is a negative-acting regulator of appressorium development that blocks cAMP/ PKA signaling downstream of cPKA when active [19]. When cAMP/PKA signaling is "on" and TOR signaling is "off", incipient appressorial development is accompanied by a single round of mitosis and autophagic cell death of the conidium [22–24]. A mature appressorium accumulates hydrostatic turgor that is directed onto a thin penetration peg, forcing it through the rice epidermis into underlying rice cells. There, *M*. *oryzae* elaborates bulbous IH and spreads undetected as a biotroph for the first four to five days of infection before necrotic lesions form [15,17]. TOR status switches from "off" to "on" as the fungus transitions from the nutrient-free leaf surface to the nutrient-rich rice cell. This is conditioned by a metabolic shift from lipid metabolism during appressorial turgor generation to glucose metabolism through the pentose phosphate pathway (PPP) during early *in planta* growth [18]. The bona fide glucose-6-phosphate (G6P) sensor trehalose-6-phosphate synthase 1 (Tps1) facilitates the shift to glucose metabolism by coordinating the genetic response to cellular glucose via an NADPH-dependent glucose-signaling pathway [25–28]. Tps1-dependent glucose metabolism via the PPP and transketolase (Tkl1) provides NADPH for antioxidation [29] and ATP to activate TOR [18]. The resulting TOR_on_ state is necessary both for the timely migration of appressorial nuclei into IH, and to promote mitosis and subsequent IH proliferation during biotrophy [18]. Thus, TOR engages one or more metabolic checkpoint in response to ATP production from glucose metabolism in order to promote fungal growth in rice cells. Where TOR regulates the cell cycle is not known.

This study was motivated by a desire to identify additional glucose signaling components in *M*. *oryzae*. By mining differential proteomic data sets from wild type (WT) and Δ*tps1* mutant strains, we identified a glucose-induced gene, *ABL1*, and determined that it functions to inhibit TOR activity in the absence of glucose. We proceeded to uncover a novel glucose-*ABL1*-TOR signaling axis connecting cellular glucose to TOR activity, cell cycle tuning, and terminal appressorial cell differentiation.

## Results

### *ABL1*, encoding an Ampk β subunit-like protein, is glucose-induced and essential for fungal pathogenicity

We identified a candidate glucose signaling factor for functional characterization by mining previously generated proteomics data [28,30] based on our rationale that unknown components of the glucose signaling pathway in *M*. *oryzae* might be responsive to glucose and dependent on Tps1 under axenic growth conditions. A protein encoded at locus MGG_00987 [31] was detected in wild type (WT) but not Δ*tps1* mycelial samples following growth on optimal 1% (w/v) glucose minimal media (GMM) with nitrate as the sole nitrogen source [28]. MGG_00987 encodes a previously uncharacterized AMP-activated protein kinase (AMPK) β subunit-like protein (Abl1). BLAST analysis suggests the 522 amino acid Abl1 protein carries an N-terminal glycogen-binding domain (GBD) that is associated with the catalytic domain of AMPK β subunits, but lacks the iteration domain carried by canonical AMPK β subunits such as the *M*. *oryzae* MoSip2 protein [32]. PSORTII analysis suggests the protein localizes to the cytoplasm.

**Fig 1:**
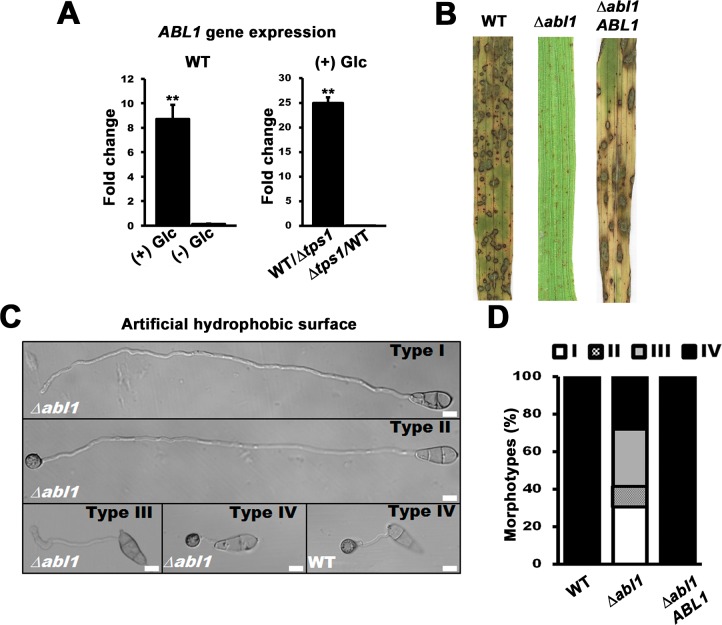
*ABL1* is essential for *M*. *oryzae* pathogenicity. (A) *ABL1* gene expression was analyzed in WT following axenic growth for 16 h in liquid minimal media with (+) or without (-) 1% (w/v) glucose (Glc) as the sole carbon source (*left panel*), and in WT relative to Δ*tps1* strains (*right panel*) after 16 h growth in 1% Glc minimal media. Ct values were normalized against the expression of the fungal *β*-tubulin gene (*TUB2*). Fold changes in *ABL1* gene expression were calculated using the 2^- (ΔΔCt)^ method. Values are statistically significant at **P<0.001 (*Student’s t-test*). Error bars are standard deviation. (B) Δ*abl1* mutant strains were non-pathogenic on susceptible CO-39 rice seedlings compared to WT. The Δ*abl1 ABL1* complementation strain was restored for fungal virulence. Images were obtained from leaves infected with 1 x 10^5^ spores/ mL and harvested at 144 hpi. (C) Appressoria formation was impaired in the Δ*abl1* mutant strain compared to WT. By 24 hpi, each germinating Δ*abl1* spore had differentiated into one of four different morphotypes: Type I, elongated germ tube with no appressoria; Type II, elongated germ tube with appressoria; Type III, irregular swelling at the germ tube tip with no appressoria; and Type IV, normal germ tube with appressoria, indistinguishable from WT. Scale bars are 10 μm. (D) Morphotype development by germinating Δ*abl1* spores was stochastic. WT and the Δ*abl1 ABL1* complementation strain only formed the Type IV morphotype. Values are the average of morphotypes scored at 24 hpi for 50 germinated spores per coverslip, repeated in triplicate for each strain with spores harvested from three different plates. Morphotype data with standard deviations are given in S1 Table.

Under axenic shake conditions, *ABL1* expression was downregulated 9-fold in WT when grown under glucose starvation conditions compared to growth on GMM with nitrate, and was downregulated 25-fold in Δ*tps1* mutant strains compared to WT on GMM with nitrate (Fig 1A). In contrast, *ABL1* gene expression was not affected by growth in GMM lacking a nitrogen source (S1A Fig). Thus, *ABL1* is expressed in response to G6P sensing by Tps1 but is not responsive to nitrogen.

To understand what role *ABL1* might play in glucose signaling, fungal physiology and/ or host infection, we deleted the *ABL1* gene from the *M*. *oryzae* genome. The resulting Δ*abl1* mutant strain sporulated like WT following growth on GMM with nitrate (S1B Fig). Despite being expressed in a glucose- and Tps1-dependent manner, *ABL1* was not required for axenic growth on GMM (S1C Fig). Moreover, whereas the yeast AMPK/Snf complex is required for the expression of glucose-repressed genes under glucose-limiting conditions [33], Δ*abl1* mutant strains grew like WT on low concentrations of glucose (S1C Fig) and on the non-preferred sugars maltose and sucrose in addition to the derepressing carbon source xylose (S1D Fig). Δ*abl1* mutant strains could also grow like WT on minimal media containing acetate as the sole carbon source (S1D Fig), which is in contrast to *M*. *oryzae* AMPK/Snf complex mutants, including Δ*mosip2*, that are impaired for lipid and acetate metabolism [32]. These results taken together show that *ABL1* is not required for carbon utilization during axenic growth and is thus functionally distinct from components of the AMPK/ Snf complex in *M*. *oryzae* and yeast [32].

Further evidence that *TPS1* is likely epistatic to *ABL1* is shown in S2A Fig. We deleted the *ABL1* gene from the genome of the Δ*tps1* mutant strain and determined that, whereas the Δ*abl1* single mutant strain could grow like WT on GMM with nitrate as the sole nitrogen source, the Δ*tps1* Δ*abl1* double mutant was, like the Δ*tps1* parental strain, nitrate non-utilizing. Δ*tps1* is poorly sporulating [25], but sporulation by the Δ*tps1* Δ*abl1* double mutant strain was completely abolished on complete media (no spores were counted after harvesting five 10 day old plates of Δ*tps1* Δ*abl1* mutant strains). Also, S2B Fig shows that, compared to WT, *TPS1* gene expression was not affected in Δ*abl1* strains following growth in GMM with nitrate. We conclude that *ABL1* acts downstream of *TPS1* and is not involved in feedback regulation of *TPS1*.

To determine if *ABL1* was important for pathogenicity, conidia of Δ*abl1* mutant strains were applied to the leaves of whole rice plant seedlings. Compared to WT and the Δ*abl1 ABL1* complementation strain, loss of *ABL1* severely attenuated fungal pathogenicity on rice leaves (Fig 1B), demonstrating *ABL1* is essential for causing rice blast disease.

### *ABL1* controls terminal appressorial cell differentiation

To understand why ablating *ABL1* impacted pathogenicity, we next examined infection-related development by Δ*abl1* mutant strains and found that the observed loss of rice infection was in major part due to impaired appressorium formation and function (Fig 1C). Spores of WT and Δ*abl1* mutant strains were applied to artificial, appressorium-inducing hydrophobic surfaces. After 24 h post inoculation (hpi), at 22°C, approximately 90% of WT spores had germinated and elaborated mature appressoria at germ tube tips (S3A Fig; Fig 1C). The remainder of WT spores either failed to germinate or formed short germ tubes without appressoria. In contrast, only approximately 34% of germinating Δ*abl1* spores formed appressoria (S3A Fig). WT rates of appressorium formation were restored in the Δ*abl1 ABL1* complementation strain (S3A Fig). Moreover, germinating Δ*abl1* spores consistently produced four distinct morphotypes (Type I-IV) by 24 hpi (Fig 1C and 1D; S1 Table). Type I Δ*abl1* morphotype (formed by 33% of spores) was distinguished as having expanded germ tubes by 24 hpi, compared to WT, with no appressoria. Type II morphotype (9% of spores) was similar but produced appressoria. Type III morphotype (33% of spores) produced undifferentiated swellings at or near germ tube tips. Type IV Δ*abl1* morphotype (25% of spores) was indistinguishable from WT. Because the majority of germinated Δ*abl1* spores (66%) did not form appressoria by 24 hpi on artificial hydrophobic surfaces (Fig 1C and 1D; S1 Table), we conclude that *ABL1* is required for appressorium formation.

On rice leaf surfaces, by 24 hpi, only approximately 20% of germinated Δ*abl1* spores formed appressoria compared to 90% for both WT and the Δ*abl1 ABL1* complementation strain (S3B Fig). Moreover, less than 20% of Δ*abl1* appressoria that formed on rice leaf surfaces were observed penetrating the cuticle into underlying epidermal cells compared to > 90% for WT and the Δ*abl1 ABL1* complementation strain (S3C Fig). Those Δ*abl1* appressoria that penetrated formed severely restricted IH that failed to grow into adjacent rice cells by 44 hpi (S3D and S3E Fig). Thus, in addition to appressorium formation, *ABL1* is required for host penetration and *in planta* growth. Subsequently, the loss of *ABL1* affected appressorial differentiation and function, increased the incidences of elongated germ tubes, and impaired biotrophic growth in rice cells.

### *ABL1* controls cell cycle progression and autophagy

In order for WT to form functional appressoria, the apical nucleus of each germinating spore undergoes one round of mitosis in the germ tube. One daughter nucleus then migrates to the incipient appressorium, the other returns to the conidium and undergoes degeneration during autophagic cell death of the spore [22–24]. To understand how these processes might be affected in Δ*abl1* mutant strains, we deleted *ABL1* from the genome of a Guy11 strain expressing histone H1 fused to red fluorescent protein (RFP) [23] to create the Δ*abl1* H1:RFP mutant strain. This strain recapitulated the original Δ*abl1* mutant phenotype such that Δ*abl1* H1:RFP strains were reduced in pathogenicity on whole rice leaves compared to the Guy11 strain carrying H1:RFP (denoted WT H1:RFP to indicate the parental background), displayed Type I-IV morphotypes in proportions similar to the original Δ*abl1* mutant strain on hydrophobic surfaces, and were impaired for growth in rice cells (S4A–S4C Fig; S1 Table).

**Fig 2:**
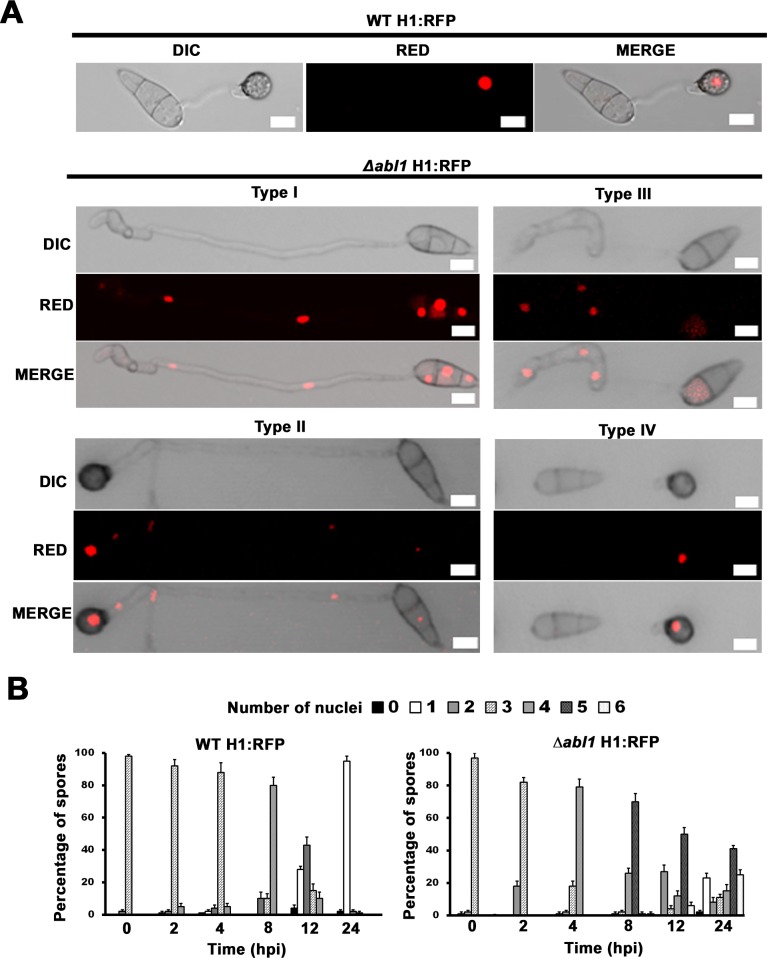
Mitosis is misregulated in Type I-III *Δabl1* mutant strain morphotypes. (A) Spores of WT H1: RFP and Δ*abl1* H1: RFP strains were inoculated at a rate of 1×10^5^ conidia/ml onto artificial hydrophobic surfaces and imaged by a Nikon A1 confocal fluorescence microscope at 24 hpi. (B) The number of nuclei carried by germinating WT H1:RFP and Δ*abl1* H1:RFP spores at the indicated time points during appressoria formation at 22°C. Mean values were calculated from three independent replicates by counting the nuclei from 100 spores per time point per strain for each replicate. Error bars are standard deviation. (A,B) All assays were performed at 22°C.

Fig 2A shows that by 24 hpi on artificial hydrophobic coverslips, germinating WT H1:RFP spores carried a single appressorial nucleus. In contrast, the majority of germinating Δ*abl1* H1:RFP spores underwent multiple rounds of mitosis in germ tubes and were impaired for conidial nuclear degeneration (Fig 2A and 2B). To quantify the affect of Δ*abl1* on mitosis and autophagy, Fig 2B shows that at 0 hpi, WT H1:RFP and Δ*abl1* H1:RFP spores carried three nuclei. By 8 hpi, germinating WT H1:RFP spores had undertaken a single round of mitosis and carried 4 nuclei (3 in the conidium and 1 in the incipient appressorium). By 24 hpi, autophagy and conidial nuclear degradation was complete and > 90% of germinated WT H1:RFP spores carried a single appressorial nucleus. In contrast, the majority of germinating Δ*abl1* H1:RFP spores had undergone one round of mitosis by 4 hpi and a second round of mitosis by 8 hpi (Fig 2B). A third round of mitosis occurred after 12 hpi in about 20% of germinating Δ*abl1* H1:RFP spores. By 24 hpi, the majority of germinated Δ*abl1* H1:RFP spores (displaying Type I-III morphotypes) carried 5 nuclei (three in the conidium and 2 in the germ tube; Fig 2A and 2B) and a significant fraction (>20%) carried 6 nuclei. These results describe how mitosis was accelerated and nuclear degradation impaired in the majority of germinating Δ*abl1* H1:RFP spores.

**Fig 3:**
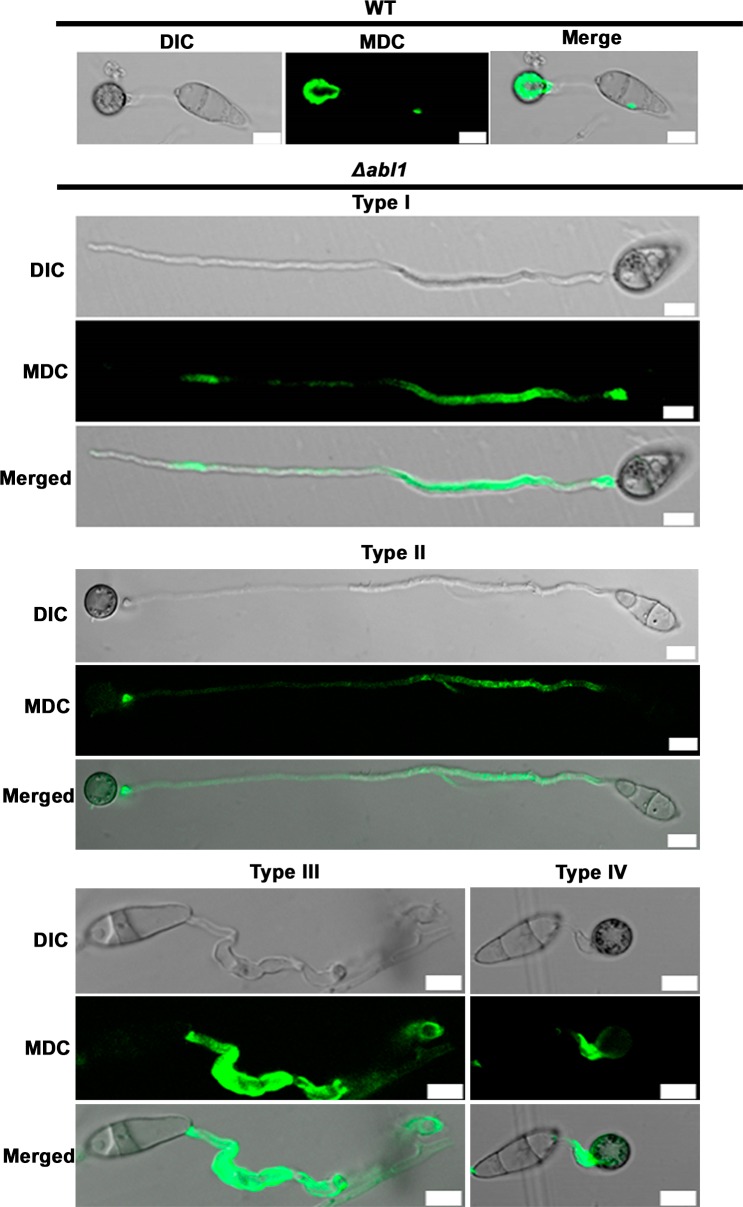
Autophagy is misregulated in *Δabl1* mutant strains. Autophagosomes were stained with 40 μM monodansylcadaverine (MDC) and examined by confocal microscopy at 24 hpi. Autophagosome distribution was misregulated in Type I-IV morphotypes of Δ*abl1* germinating spores compared to WT.

Additional evidence that autophagy was misregulated in Δ*abl1* mutant strains is shown in Fig 3. Monodansylcadaverine (MDC) staining at 24 hpi shows autophagosomes were concentrated in the appressorium of WT but were dispersed throughout the germ tube in Δ*abl1* mutant strains. Moreover, glycogen mobilization is an important feature of appressorium morphogenesis [34]. S5 Fig shows how glycogen mobilization, like autophagy, was severely impaired in Type I-III morphotypes of Δ*abl1* mutant strains compared to WT and was delayed in Type IV morphotypes.

### *ABL1* inactivates TOR signaling during terminal appressorial cell differentiation

**Fig 4:**
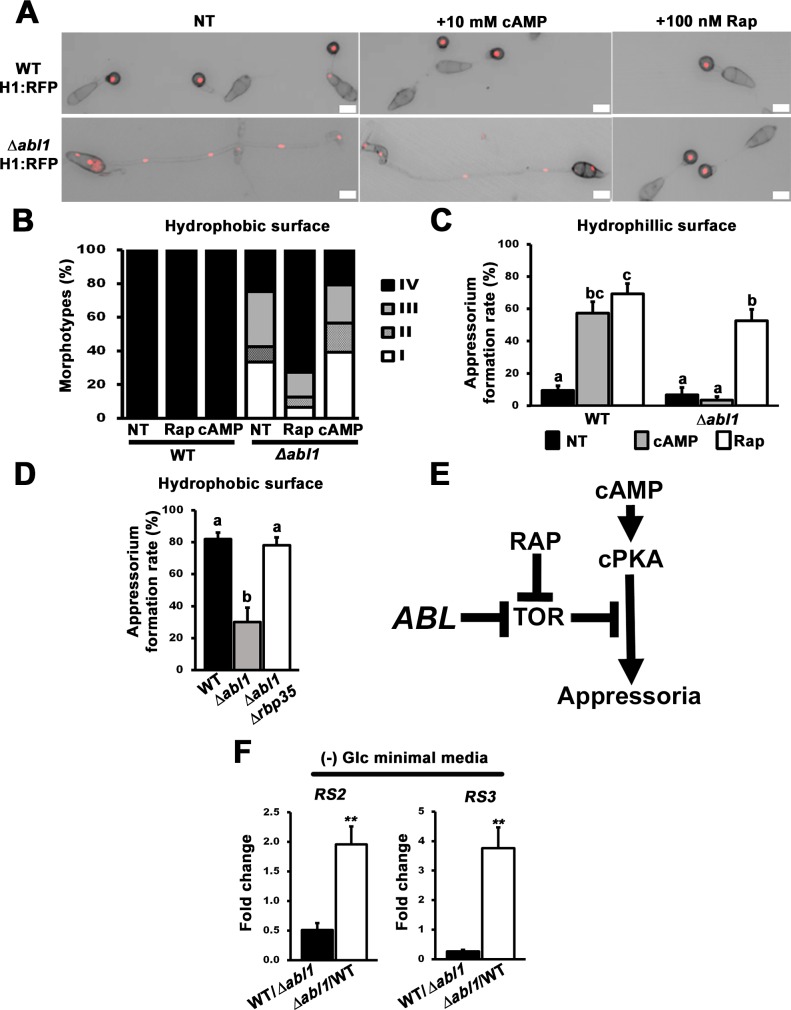
Active TOR-signaling in Δ*abl1* mutant strains acts downstream of cPKA to promote mitosis and impair both autophagy and appressorium formation. (A) Rapamycin (Rap) treatment, but not cAMP, restored appressorium formation and autophagy to Δ*abl1* H1:RFP strains when viewed at 24 hpi. All panels are merged DIC and fluorescence images. Scale bars are 10 μm. (B) Rap but not cAMP treatment increased Type IV and decreased Type I morphotypes in Δ*abl1* H1:RFP strains at 24 hpi. Values are the average of morphotypes scored at 24 hpi for 50 germinated spores per coverslip, repeated in triplicate for each strain and treatment with spores harvested from three different plates. Morphotype data with standard deviations are given in S1 Table. (C) Rap but not cAMP treatment induced Δ*abl1* appressorial development on hydrophilic surfaces. (D) Appressorium formation rates of the Δ*abl1*Δ*rbp35* double mutant strain were significantly increased compared to Δ*abl1* single mutant strains on hydrophobic artificial surfaces. (E) *ABL1* promotes appressorium formation by inhibiting TOR. (F) *RS2* and *RS3* ribosomal gene expression was quantified in WT and Δ*abl1* mutant strains following 16 h of growth in minimal media without glucose (-Glc). Relative fold changes in gene expression were calculated using the 2^- (ΔΔCt)^ method with *β*-tubulin (*TUB2*) as the internal control gene. Values are statistically significant at **P<0.001 (*Student’s t-test*). Values were calculated in triplicate from two independent biological replications. (C-D) Appressorium formation rates were determined at 24 hpi from 50 spores per hydrophilic slide or hydrophobic coverslip, repeated in triplicate. Bars with different letters are significantly different (P ≤ 0.05). (A-C) NT = no treatment. (C-D,F) Error bars are the standard deviation.

We next discovered that *ABL1* controls appressorium development, mitosis and autophagy via the TOR signaling pathway. This was achieved by determining the relationship of *ABL1* to characterized signaling pathways that regulate appressorial morphogenesis. Loss of the cAMP/PKA signaling pathway [21], or activation of TOR-signaling to inhibit the cAMP/PKA signaling pathway downstream of cPKA [19], abolishes appressorium formation. Appressorium formation can be remediated in strains carrying mutations upstream of cPKA by cAMP treatment, and in TOR-activated mutants by treatment with the specific TOR kinase inhibitor rapamycin (Rap). Fig 4A and 4B, S4B Fig and S1 Table show that Rap treatment, but not treatment with cAMP, induced autophagy and morphologically normal (Type IV) appressorium formation in Δ*abl1* and Δ*abl1* H1:RFP mutant strains. These data strongly suggest TOR signaling is inappropriately activated in Δ*abl1* mutant strains during germination to block cAMP/ PKA signaling downstream of cPKA. Consistent with this notion, Rap and cAMP both induced appressorium formation in WT on non-inductive hydrophilic surfaces, as noted previously [19], but only Rap induced appressorium formation in Δ*abl1* strains on hydrophilic surfaces (Fig 4C).

Evidence that *ABL1* functions as an upstream inhibitor of TOR signaling is shown in Fig 4D. Rbp35 is an *M*. *oryzae* RNA-binding protein involved in processing RNA transcripts essential for host infection [35]. Δ*rbp35* mutant strains form appressoria but are downregulated for TOR pathway activity [35]. We reasoned that if perturbed appressorium formation in Δ*abl1* mutant strains was due to constitutively active TOR signaling, then the loss of *RBP35* in Δ*abl1* mutant strains might restore appressorium formation in this mutant background. Fig 4D shows that Δ*abl1* Δ*rbp35* double mutant strains had WT appressorial formation rates on hydrophobic surfaces. Fig 4E summarizes the deduced relationships between *ABL1*, TOR signaling, the cAMP/PKA pathway, and appressorium formation.

Additional and direct evidence that TOR is inappropriately activated in Δ*abl1* mutant strains is shown in Fig 4F. TOR signaling controls ribosomal gene expression [36], and the ribosomal genes *RS2* and *RS3* are expressed in *M*. *oryzae* when TOR signaling is active and downregulated when TOR is inactive [18]. Following growth under glucose starvation conditions, we observed by qPCR that *RS2* and *RS3* were significantly (**P<0.001) more highly expressed in Δ*abl1* mutant strains than in WT.

Taken together, our results indicate *ABL1* acts as a novel upstream inhibitor of TOR function to promote appressorium formation, induce autophagy and arrest mitosis during appressorium morphogenesis. Conversely, activated TOR signaling in Δ*abl1* mutant strains inhibits appressorium formation downstream of cAMP/PKA, promotes mitosis and germ tube elongation, and abolishes autophagy.

### *ABL1*-dependent cell cycle arrest at G2/M induces appressorium morphogenesis

**Fig 5:**
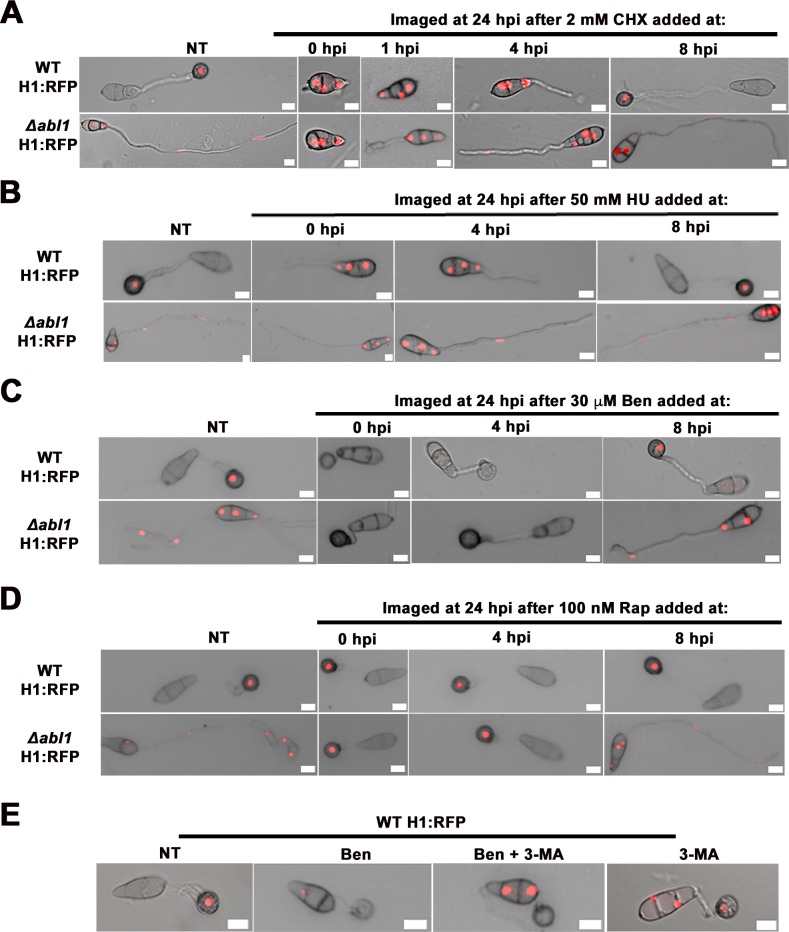
*ABL1*-TOR dependent G2/M arrest induces autophagy and appressorium formation. (A) Cyclohexamide (CHX) and (B) hydroxyurea (HU) treatment induced G1 and S-phase arrest (respectively) but did not induce appressoria formation or autophagy in Δ*abl1* H1: RFP strains. (C) Benomyl (Ben) treatment led to cell cycle arrest at G2/M phase and induced appressoria formation and autophagy in Δ*abl1* H1: RFP strains if added before 8 hpi. (D). Rapamycin induced appressoria formation and autophagy in Δ*abl1* H1: RFP strains if added before 8 hpi. (E) Dual treatment with Ben and the autophagy inhibitor 3-MA indicates autophagy is induced following G2 arrest. Treatment with 3-MA alone impaired mitosis during appressorium formation. (A-E) All panels are merged DIC and fluorescence images. Treatments were added at the indicated times, at 22°C, and viewed at 24 hpi. Scale bars are 10 μm.

In yeast and mammalian cells, TOR inactivation terminates protein synthesis resulting in G1 arrest [12] and autophagy [37]. To determine whether G1 arrest was necessary and sufficient to account for the induction of appressorium formation and autophagy in Rap treated Δ*abl1* mutant strains, we treated germinating spores of WT H1:RFP and Δ*abl1* H1:RFP with cycloheximide (CHX). Similar to TOR inactivation, CHX induces G1 arrest by inhibiting protein synthesis and reducing the translation of cyclins [12, 38]. Spores of each strain were applied to coverslips at 22°C, treated with CHX at the indicated time points, and viewed at 24 hpi (Fig 5A and S2 and S3 Tables). Adding CHX at 0 hpi abolished germination in both strains when viewed at 24 hpi, indicating that protein synthesis is a requisite for spore germination in *M*. *oryzae*. Note that adding Rap at 0 hpi does not affect germination (Fig 4A), indicating TOR_off_ does not arrest in this first G1 phase. CHX treatment at 1 hpi shows Δ*abl1* H1:RFP is accelerated for germination compared to WT H1:RFP. Adding CHX to germinating WT H1:RFP spores at 4 hpi prevented mitosis and appressorium formation, indicating WT apical conidial nuclei have not passed START and are not committed to mitosis by 4 hpi. In contrast, adding CHX to germinating Δ*abl1* H1:RFP spores at 4 hpi after one round of mitosis (or adding CHX to Δ*abl1* H1:RFP spores at 8 hpi after the second round of mitosis) arrested the cell cycle in the next G1 phase but did not induce appressorium formation. These results demonstrate, firstly, that Δ*abl1* H1:RFP apical conidial nuclei have passed START and exited the first G1 phase by 4 hpi, consistent with accelerated mitosis in Δ*abl1* strains (Fig 2A and 2B). Secondly, these results provide strong evidence that G1 arrest alone is not sufficient to induce appressorium formation and autophagy in Δ*abl1* mutant strains.

Arresting the cell cycle at S-phase by adding the DNA synthesis inhibitor hydroxyurea (HU) at 0 hpi also did not induce appressorium development or autophagy in WT or Δ*abl1* mutant strains (Fig 5B; S2 and S3 Tables). Note that HU treatment at 0 hpi permitted spore germination, but nuclear migration into the germ tube did not occur for either strain indicating that an S-phase checkpoint has to be cleared for apical nuclei to migrate into the germ tube before mitosis. Moreover, in WT, this S-phase checkpoint has to be cleared in order for appressorium formation to be induced [22]. However, adding HU to germinating Δ*abl1* H1:RFP spores at 4 hpi arrested mitosis at the second S-phase after the first round of mitosis but did not induce appressoria or autophagy. Cell cycle progression through DNA replication is therefore necessary but not sufficient to induce appressorium formation or autophagy.

We next treated spores with the G2 inhibitor benomyl (Ben). Fig 5C and S2 and S3 Tables show that Ben treatment of germinating Δ*abl1* H1:RFP spores induced the formation of aberrant appressorium formation with short germ tubes and complete nuclear degeneration if added at 0 hpi before the first G2 phase in Δ*abl1* strains, or at 4 hpi before the second G2 phase following the first round of mitosis (Fig 2B), but not if added at 8 hpi (after which further mitosis is delayed or mostly arrested in this mutant strain, Fig 2B). These results strongly imply the novel proposition that *ABL1*-dependent cell cycle arrest at the G2/M checkpoint is required for appressorium and autophagy induction.

To determine if the appressorium-inducing G2 arrest occurred via TOR, we treated Δ*abl1* H1:RFP spores with Rap at different time points and viewed the effect on appressorium morphogenesis at 24 hpi. Fig 5D and S2 and S3 Tables show that treating germinating Δ*abl1* H1:RFP spores with Rap before the first (at 0 hpi) or second (at 4 hpi) G2 phase, but not later (at 8 hpi), induced appressorium formation and autophagy and progressed the cell cycle to G1 arrest, resulting in normal appressorium formation. Treating Δ*abl1* H1:RFP spores with Rap at later time points arrested mitosis in G1 (confirming *ABL1* acts via TOR in G1 arrest) but did not induce appressoria formation or autophagy. Thus, TOR_off_ is essential for G2 arrest and the induction of appressorium formation, autophagy and cell cycle progression to G1.

### Cell cycle re-progression after G2/M arrest requires autophagy

The previous results suggested that a G2 arrest followed by cell cycle re-progression to G1 are required for proper appressorial development by untreated WT or Rap treated Δ*abl1* spores. Similarly in yeast, reduced TOR activity under nutrient limiting conditions or following Rap treatment leads to cell cycle arrest at the G2/M transition. This is followed by the induction of autophagy. Autophagy-dependent activation of TOR then restarts mitosis and allows cell cycle re-progression to G1 arrest [11, 14]. To determine if cell cycle re-progression to G1 after G2 arrest was dependent on autophagy in *M*. *oryzae*, we treated spores with the autophagy inhibitor 3-Methyladenine (3-MA). Compared to single Ben treatment, which resulted in complete nuclear degeneration, treatment with both Ben and 3-MA inhibited autophagy of conidial nuclei (Fig 5E). This demonstrated that autophagy and appressorium induction coincided with G2 arrest, but autophagy was not required for the induction of appressorial development. Treatment with 3-MA alone resulted, by 24 hpi, in apical nuclei migrating into the incipient appressorium without undergoing mitosis, indicating autophagy is required for the completion of the cell cycle following G2 arrest. When considered along with evidence showing G1 arrest alone does not induce autophagy in Δ*abl1* H1:RFP strains (Fig 5A and 5D), the data in Fig 5E supports the notion that *ABL1*-TOR dependent G2/M arrest co-induces appressorium development and autophagy with the latter process required for cell cycle re-progression to G1 arrest and the production of functional appressoria.

### *ABL1* connects glucose signaling with TOR activity

**Fig 6:**
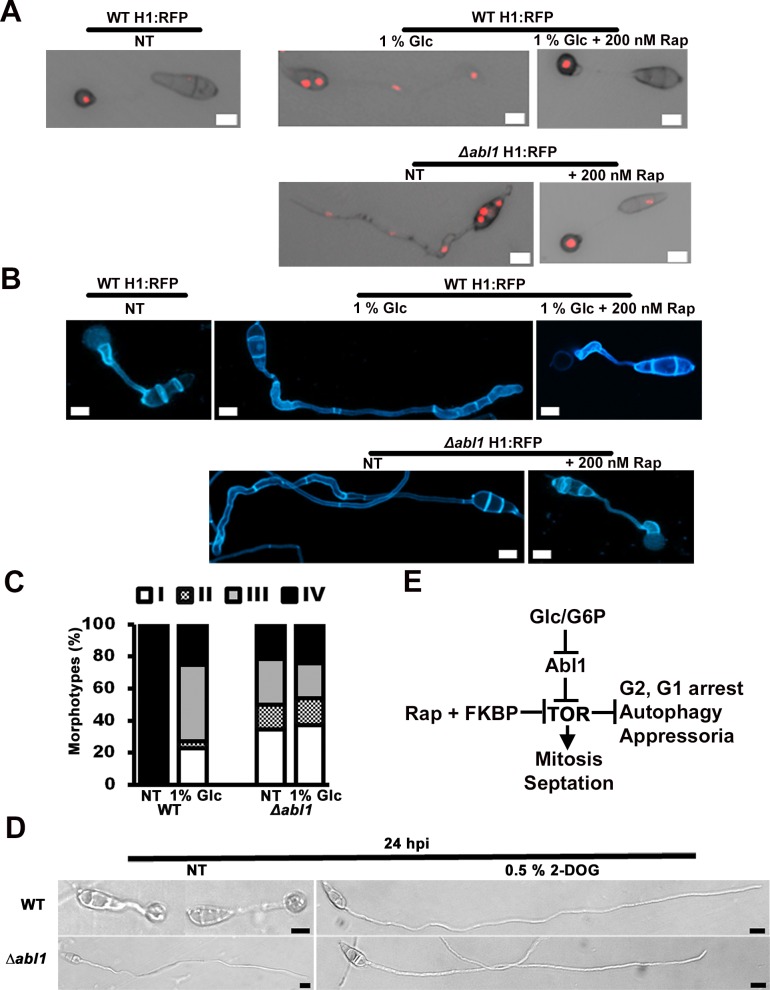
Glucose-*ABL1-*TOR signaling regulates terminal appressorial cell differentiation. (A) Adding 1% (w/v) glucose (Glc) to WT H1: RFP spores phenocopied untreated Δ*abl1* H1: RFP strain development at 24 hpi on hydrophobic surfaces and was remediated by Rap. (B) 0.02% (w/v) calcafluor white staining shows Glc treatment increased septation by germinated WT spores and phenocopied the increased septation observed in untreated Δ*abl1* H1: RFP strains. Septation defects were remediated in both cases by Rap. (A, B) All panels are merged DIC and fluorescence images taken at 24 hpi. Appressoria developed at 22°C. Scale bars are 10 μm. (C) Glc treatment of WT spores provoked stochastic morphotype development with frequencies similar to those observed for untreated Δ*abl1* H1: RFP strains. Glc treatment of Δ*abl1* H1: RFP spores did not affect morphotype frequency compared to no treatment (NT) controls. Values are the average of morphotypes scored at 24 hpi for 50 germinated spores per coverslip, repeated in triplicate for each strain with spores harvested from three different plates. Morphotype data with standard deviations are given in S1 Table. (D) Spores were treated with 1% (w/v) 2-deoxyglucose (2-DOG) at 0 hpi and imaged at 24 hpi. Scale bars are 10 μm. (E) Model of relationship between glucose-*ABL1*-TOR signaling and the cell cycle, autophagy and appressorium formation.

Considering *ABL1* is a glucose-responsive gene (Fig 1A), we next sought to determine the relationship between glucose and the *ABL1*-TOR signaling pathway described above. Treating germinating WT H1:RFP spores with 1% (w/v) glucose (Glc) phenocopied Δ*abl1*. Specifically, the development of germinating WT H1:RFP spores treated with Glc at 0 hpi was indistinguishable from untreated Δ*abl1* H1:RFP spores by 24 hpi: both produced long germ tubes, most without appressoria (S1 Table), underwent multiple rounds of mitosis and were abolished for autophagy (Fig 6A). Adding Rap to Glc treated WT spores at 0 hpi overrode the glucose mitotic proliferation signal and, like Δ*abl1* spores treated with Rap only, restored appressorium formation and autophagy by 24 hpi. In addition, septation was misregulated in both Glc treated WT H1:RFP strains and untreated Type I-III Δ*abl1* H1:RFP morphotypes (but not Type IV), and these developmental defects were also remediated by Rap treatment (Fig 6B and S6A Fig). These results place glucose signaling, like *ABL1*, upstream of TOR.

S6B Fig confirms that glucose signaling is transmitted via TOR. Although Rap treatment could remediate appressorium formation by glucose treated WT spores, it could not induce appressorium formation by glucose treated Δ*fpr1* spores. *FPR1* encodes FKBP12, a component of the FKBP-rapamycin complex that physically and specifically interacts with TOR to inhibit its activity; loss of FKBP12 renders Δ*fpr1* mutant strains insensitive to rapamycin [19].

By 24 hpi, glucose treated WT spores produced morphotypes (Fig 6C; S1 Table) and nuclei (S6C Fig) in similar proportions and numbers to those observed for untreated Δ*abl1* spores. Mitosis was also accelerated in glucose treated germinating WT spores (S6D Fig). However, glucose treatment did not affect the phenotype of germinating Δ*abl1* spores compared to untreated Δ*abl1* controls (Fig 6C; S6C Fig; S1 Table). These results indicate that phenotypes resulting from glucose treatment or *ABL1* deletion are not additive and confirm that the loss of *ABL1* mimics glucose treatment during appressorial differentiation, thus placing glucose signaling upstream of *ABL1*.

Although *ABL1* is expressed in the presence of glucose in response to G6P sensing by Tps1 (Fig 1A), a functional *ABL1* gene is required to inhibit TOR in the absence of glucose (Fig 6A). To resolve this paradox, we considered that the *ABL1* gene was expressed in the presence of glucose when its protein product was not required in order to prime the cell with Abl1 in order to rapidly inactivate TOR and delay mitosis if glucose became limiting. If so, we predicted that although *ABL1* expression was inducted by glucose and downregulated in glucose starvation conditions, the Abl1 protein should be detectable under both conditions. In support of this notion, S7A Fig shows that Abl1 protein was detected in samples of mycelia grown either in either the presence or the absence of glucose. We complemented Δ*abl1* with the *ABL1* gene, under its native promoter, that was fused at the 3’ end to the gene encoding green fluorescent protein (GFP). Δ*abl1 ABL*^*GFP*^ strains expressing the Abl1^GFP^ fusion protein were, like the complementation strain discussed above, restored for pathogenicity and appressorium formation, indicating Abl1^GFP^ was functional. However, Abl1^GFP^ fluorescence was not detected above background autofluorescence, suggesting the protein was present in low amounts or in a configuration that precluded detection by confocal microscopy. Nonetheless, probing Western Blots with anti-GFP identified bands of Abl1^GFP^ at the predicted 63 kDa size in protein samples extracted from mycelia grown in CM for 48 h before switching to CM with or without glucose for 2 h (S7A Fig). Moreover, when quantified relative to the loading control α-Tubulin, Abl1^RFP^ was more abundant following growth in glucose-limiting (-Glc) conditions than in glucose-sufficient (+Glc) conditions (S7B Fig). When considered with Fig 1A, these results suggest *ABL1* is expressed, and the Abl1 protein accumulates, in the presence of glucose, but the protein remains abundant when glucose is depleted and *ABL1* expression is downregulated. Additional evidence that the Abl1 protein is functional under glucose-limiting conditions when *ABL1* gene expression is downregulated is shown in S7C,D. After 2 h growth in liquid media lacking glucose (following 48 h growth in CM with glucose), the mycelia of Δ*abl1* H1:RFP strains carried significantly more nuclei (p ≤ 0.05) than WT H1:RFP mycelia. Also, consistent with increased mitosis in Δ*abl1* mutant strains, the mass of dry weight mycelia was also increased in Δ*abl1* H1:RFP mutant strains under glucose starvation conditions compared to WT (S7E Fig). Thus, the Abl1 protein functions as a brake on mitosis and growth when cellular glucose becomes limiting.

To be an efficient brake, we next predicted that although *ABL1* expression was induced by glucose, the Abl1 protein would be maintained in an inactive state by glucose or a downstream metabolite. Fig 6D shows how treatment of WT spores with the glucose analogue 2-deoxyglucose (2-DOG) mimicked glucose treatment or the loss of *ABL1* function by inducing long germ tubes and inhibiting appressorium formation. 2-DOG is phosphorylated to the non-metabolizable G6P analogue 2-DOG-P, resulting in glycolysis inhibition and ATP depletion. This suggests glucose or G6P, but not downstream metabolites, activate TOR by inactivating Abl1 and confirms Abl1 as a glucose-responsive, negative-acting regulator of TOR.

Together, we can confidently propose that glucose or G6P (directly or indirectly) inhibits Abl1 function in order to activate TOR and drive mitosis. *ABL1* deletion activates TOR in the absence of glucose signaling (Fig 6E). Abl1 thus transmits inhibitory glucose depletion signals to TOR.

### Relationship between Tps1 and Abl1 during appressorium formation

Δ*tps1* strains are non-pathogenic on rice plants [39]. Because Tps1 was shown to be epistatic to *ABL1* (S2A Fig), and necessary for *ABL1* gene expression (Fig 1A), we reasoned that treating Δ*tps1* mutant strains with rapamycin (or overexpressing *ABL1*) might improve virulence. However, although defects in Δ*tps1* appressoria function have been reported in the literature [39], S2C Fig shows that on rice leaf surfaces, Δ*tps1* mutant strains formed appressoria at the same rate as WT regardless of whether or not spores were first treated with Rap. Moreover, S2D Fig shows that Δ*tps1* appressoria penetrated at the same rate as WT regardless of Rap treatment. Nonetheless, S2E Fig shows that regardless of Rap treatment, Δ*tps1* growth was attenuated in the host rice cell. This initially suggested to us that reduced *ABL1* expression in Δ*tps1* strains was not equivalent to the loss of *ABL1* function in Δ*abl1* strains. However, we urge caution with the interpretation of these results. Δ*tps1* mutant strains, unlike Δ*abl1* strains, are severely attenuated for sporulation rates [25]. For WT and Δ*abl1* mutant strains, we required only one CM plate to provide enough spores for inoculating rice leaf sheaths. In contrast, 200 CM plates we were needed to provide enough Δ*tps1* spores to perform the rice leaf sheath assays. This prolonged harvesting of Δ*tps1* spores might bias our observations towards Type IV Δ*tps1* morphotypes, or, despite extensive washing of the spores, could introduce a contaminating metabolite from the media that enhances appressorium function in Δ*tps1* relative to Δ*abl1* mutant strains. Thus, while the genetic connection between G6P sensing, *TPS1* and *ABL1* under axenic shake conditions is clear (Fig 1A), the poor sporulation rate of Δ*tps1* might affect our study of the Tps1 *–*Abl1 interaction on rice surfaces. Fully exploring the relationship between Tps1, Abl1 and fungal virulence is a challenge that will require developing a system to induce sporulation in Δ*tps1* mutant strains, an ongoing endeavor of our group.

### Glucose signaling engages metabolic checkpoints at G2 and G1 via TOR

**Fig 7:**
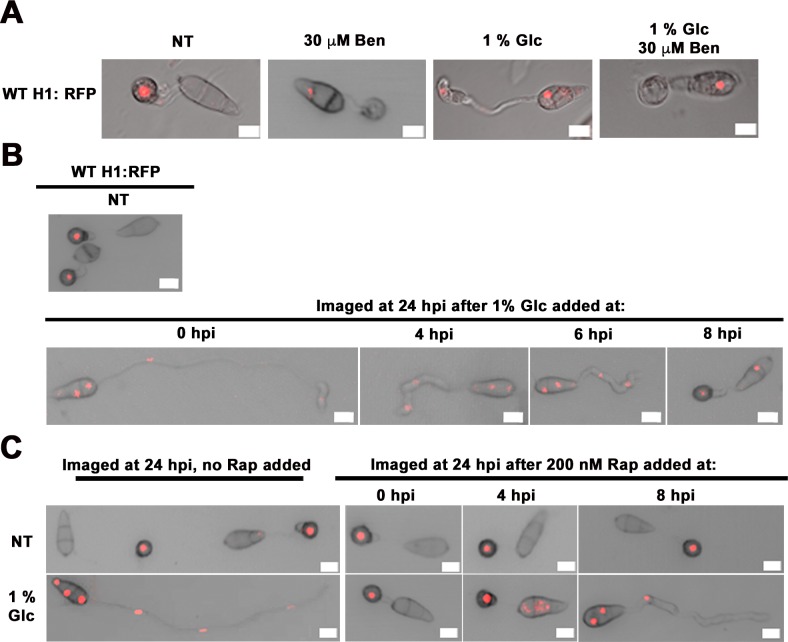
Glucose acts at G2 and G1. (A) Dual Ben + Glc treatment induced appressorium formation and autophagy. (B) Glc added before but not after G2 abolished appressorium formation and autophagy. (C) Rap added to Glc treated spores induced appressorium formation and autophagy if added before but not after G2. Adding after G2 arrested at the next G1. Images were taken at 24 hpi.

We next confirmed that glucose controlled mitosis at G2 and G1 via TOR. Fig 7A shows that Ben treatment induced appressorium formation in glucose treated WT H1:RFP spores. This places G2 arrest downstream of glucose signaling. Fig 7B shows that when glucose was added to germinating WT H1:RFP spores before but not after G2, appressorium formation and autophagy were abolished, and mitosis accelerated. Similarly, Fig 7C shows that Rap added before G2 to glucose-treated germinating spores could override the glucose signal and induce appressorium formation and autophagy, but adding Rap after G2 at 8 hpi prevented further rounds of mitosis (by arresting at G1) but did not induce appressoria formation or autophagy. Together, these results indicate glucose activates TOR to prevent G2 and G1 arrest. This work supports the notion that in the absence of glucose, G2 arrest is a commitment step towards appressorium formation that is succeeded by autophagy and cell cycle re-progression to G1/G0 arrest.

### Glucose-*ABL1*-TOR signaling controls cell cycle quiescence at G1/G0 in terminally differentiated appressorial cells

**Fig 8:**
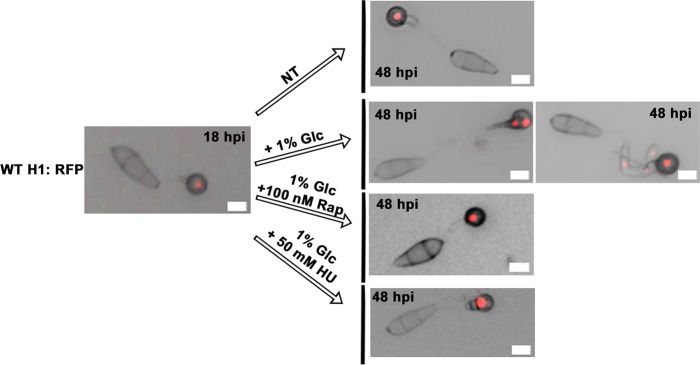
Glucose-*ABL1*-TOR signaling maintains reversible cell cycle quiescence of appressorial nuclei at G1/G0. Treatments were added to coverslips at 18 hpi. All panels are merged DIC and fluorescence images taken at 24 or 48 hpi. Scale bars are 10 μm. NT = no treatment.

Rather than being arrested at G1, we next discovered that appressorial nuclei were maintained in a reversibly quiescent/ G0 state dependent on the glucose-*ABL1*-TOR signaling axis. This was determined by asking if treating terminally differentiated appressoria with glucose activated TOR and re-started the cell cycle. We added 1% (w/v) Glc (or water as a no treatment control) to WT H1:RFP strains at 18 hpi. At this time point, autophagic degeneration of conidial nuclei was complete and a single daughter appressorial nucleus remained (Fig 8). When viewed again at 48 hpi, no further rounds of mitosis were evident in untreated samples, indicating terminally differentiated appressorial cells had exited the cell cycle. In contrast, by 48 hpi, samples treated with glucose at 18 hpi demonstrated several phenotypes, including hyphal branching from the germ tube, that were all characterized by carrying more than one nucleus. These results demonstrate how appressorial nuclei can re-enter mitosis in the presence of glucose. To confirm that the observed glucose-dependent cell cycle re-entry acted via TOR, Fig 8 shows that simultaneous treatment with glucose and Rap at 18 hpi did not induce mitosis in appressorial nuclei by 48 hpi. To confirm that appressorial nuclei were held in a quiescent state at G1/G0 (rather than at G2/M) Fig 8 shows that simultaneous treatment with glucose and HU at 18 hpi also did not induce mitosis in appressorial nuclei by 48 hpi. Thus, glucose treatment reversed the G1/G0 quiescent state of terminal appressorial cell nuclei and induced mitosis. These results are consistent with our understanding that, following transition into the host, ATP production from glucose metabolism activates TOR and promotes fungal mitosis in rice cells [18]. These results are also interesting in light of Fig 7B which shows that adding glucose at 8 hpi did not prevent mitotic arrest by 24 hpi, suggesting both that appressorial nuclei become committed to exiting the cell cycle before mitosis and following G2 arrest, and that passage through G0 and back into the cell cycle in the presence of glucose requires more time than the 16h between 8 hpi and 24 hpi (Fig 7B). This would be consistent with reports indicating that the transition from G0 to S phase is longer than the transition from G1 to S phase in mammalian cells [40], supporting the notion that appressorial nuclei have exited the cell cycle into a resting quiescent/ G0 state.

### Models of cell cycle progression in germinating spores

To further resolve cell cycle phase boundaries during WT and Δ*abl1* mutant strain spore germination, S8A Fig shows that, whereas Ben treatment of germinating WT H1:RFP spores at 8 hpi permitted the formation of normal appressoria populated with a single appressorial nucleus (Fig 5C), Ben treatment at 6 hpi resulted in aberrant appressorium formation and complete nuclear degradation. This indicates that the G2 checkpoint in germinating WT spores occurs between 6 hpi and 8 hpi (S8A Fig). In addition, to more accurately determine when the first G1 phase is completed in germinating Δ*abl1* H1:RFP spores, spores were treated with CHX at 2 hpi and 3 hpi. S8B Fig shows that in germinating Δ*abl1* mutant strains, G1 is completed after 2 hpi and before 3 hpi.

**Fig 9:**
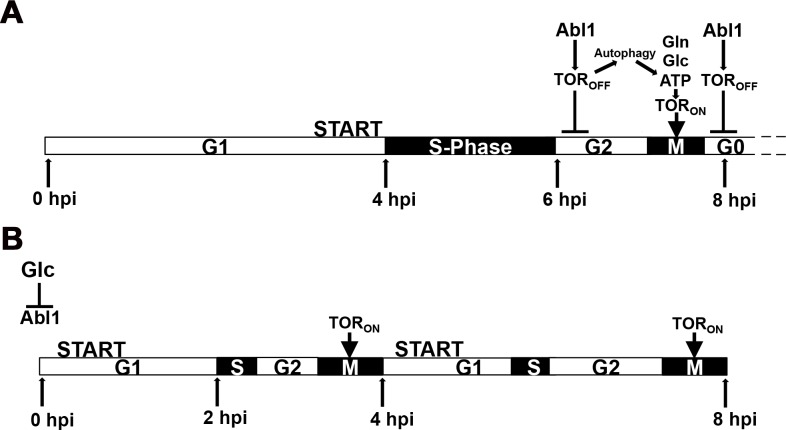
Glucose-*ABL1*-TOR signaling mediates cell cycle progression and quiescence during appressorium formation. Models of cell cycle tuning by the glucose-*ABL1*-TOR signaling axis during WT appressorial cell differentiation under (A) nutrient-free conditions and (B) in the presence of glucose or the absence of *ABL1* function. Cell cycle phase lengths were determined from data in Figs 2, 4 and 6 and S8 Fig. Autophagy at G2 in (A) might yield glutamine (Gln), glucose (Glc) or ATP to activate TOR.

When all our data are considered together, we propose the models in Fig 9 to account for cell cycle progression during spore germination and appressorium formation. Fig 9A shows that in untreated WT spores, or rapamycin treated WT and Δ*abl1* spores, TOR_off_ arrests the cell cycle at G2 after 6 hpi resulting in the induction of appressorial formation and, independently, autophagy. Consistent with reports in yeast [11,14], an autophagy-mediated TOR_on_ state then enables cell cycle re-progression through mitosis to TOR_off_ and, in *M*. *oryzae*, glucose-dependent reversible quiescence at G1/G0 in mature appressoria (Fig 9A). Consistent with the importance of TOR_on_ for mitosis, we note that active TOR prevents M phase delay in yeast [41]. Fig 9B shows that in Δ*abl1* or glucose-treated spores, constitutive TOR_on_ results in multiple rounds of accelerated mitosis during germination (compared to untreated WT spores). The loss of cell cycle arrest under these conditions results in the loss of appressorial development and autophagy.

### The role of intracellular glutamine in cell cycle progression

Autophagy at G2 arrest can liberate TOR activating substrates such as glucose (through gluconeogenesis), ATP, and glutamine [14,42] (Fig 9A). Consistent with the notion that autophagy-liberated substrates promote TOR_on_, S9A Fig shows how treatment of WT spores with exogenous glutamine partially impaired appressorium formation and resulted in elongated germ tubes. S9B Fig shows that glutamine treated WT spores displayed the Type I, II and IV morphotypes observed for germinating Δ*abl1* and glucose treated WT spores (Fig 6C), but in different proportions, and absent Type III. This suggests glutamine and glucose might activate TOR by different pathways and/or with different efficiencies and/ or at different stages of the cell cycle.

The large proportion of glutamine treated germinating spores displaying the Type IV morphotype by 24 hpi (S9B Fig), compared to glucose treatment (Fig 6C), might reflect reduced uptake of glutamine from the spore suspension due to its poor solubility in water. To better understand the role of intracellular glutamine in cell cycle progression, appressorium formation and autophagy, we turned our attention to the Δ*asd4* mutant strain. *ASD4* encodes a GATA-family transcription factor involved in regulating the expression of genes required for nitrogen assimilation and glutamine metabolism through the GS-GOGAT pathway. Loss of *ASD4* in Δ*asd4* mutant strains misregulated target genes resulting in elevated intracellular glutamine levels that, during Δ*asd4* spore germination, activated TOR and prevented appressorium formation [19]. Appressorium formation was restored in Δ*asd4* mutant strains by the treatment of germinating spores with rapamycin. We show here that in addition to abolished appressorium formation, Δ*asd4* mutant strains were also impaired for autophagy as determined by MDC staining at 24 hpi (S10A Fig); were impaired for glycogen mobilization (S10B Fig); demonstrated increased septation in germ tubes as determined by calcofluor white staining at 24 hpi (S10C Fig); and carried nuclei in germ tubes at 24 hpi that might result from additional rounds of mitosis, as determined by SYTO green fluorescent nucleic acid staining (S10D Fig). However, while the phenotypes of Δ*asd4* and Δ*abl1* are similar, they are not equivalent because Δ*asd4* strains never exhibit Type IV or Type II morphotypes (S10 Fig; [19]). Our interpretation of these results is that glutamine, like glucose, is a modulator of TOR activity but acts on TOR at a different stage of the cell cycle, as predicted in Fig 9A. Consequently, glutamine liberation following autophagy in germinating WT spores might rescue the cell cycle delay at G2/M.

## Discussion

TOR kinase orchestrates conserved growth-signaling pathways in eukaryotes. In addition to critical roles in human pathologies [2], the TOR signaling pathway has recently emerged as a central regulator of rice infection by the devastating blast fungus *M*. *oryzae* [18, 19]. Elucidating the regulation and scope of TOR activity has wide ranging implications for our understanding of cell growth and development, but it is not well known how -or to what end—cellular glucose controls TOR, or how TOR receives inhibitory signals. Here, we uncovered a novel glucose-*ABL1*-TOR signaling axis that mediates cell cycle tuning in response to glucose. This was achieved by first identifying *ABL1* as a previously unknown glucose signaling component and subsequently characterizing its function as a negative-acting TOR regulator required for terminal appressorial cell differentiation.

### Insights on glucose control of appressorial cell differentiation

During WT spore germination at 22°C, *ABL1* mediates TOR_off_ to arrest the cell cycle at G2, resulting in the induction of appressorial formation and, independently, autophagy. Autophagy can liberate TOR activating substrates such as glutamine. An autophagy-mediated TOR_on_ state then enables cell cycle re-progression through mitosis to TOR_off_ and glucose-dependent reversible quiescence at G1/G0 in mature appressoria (Fig 9A). Consistent with the notion that autophagy-liberated substrates promote TOR_on_, we noted that elevated intracellular glutamine levels (resulting from impaired glutaminolysis) inhibited appressorium formation in *M*. *oryzae* Δ*asd4* mutant strains by constitutively activating TOR [19]. However, Δ*asd4* [19] and glutamine treated WT spores did not display the full range of morphotypes associated with glucose treatment or *ABL1* loss, suggesting glucose and glutamine converge to activate TOR at different times or via different pathways that remain to be resolved.

Inactivating *ABL1* function through ablation or glucose treatment compromised TOR inhibition and caused constitutive TOR_on_ status in germinating spores (Fig 9B). G2 and G1 metabolic checkpoint arrests were subsequently eliminated under these conditions resulting in shortened G1/S and G2/M phases and multiple, accelerated rounds of mitosis. G1 was completed around 2 hpi, indicating TOR_on_ rapidly advances G1 to START, the point beyond which cells are committed to mitosis [43]. G2/M was completed by 4 hpi without inducing autophagy or appressorium formation. G2/M is the primary cell size control point in fission yeast [44], suggesting a short G2 phase might obviate appressorial elaboration by preventing extended germ tube tip growth. Appressorium formation could be remediated in Δ*abl1* mutant strains by rapamycin treatment or by deleting *RBP35*. Thus, biochemical and genetic data provide strong evidence that *ABL1* controls TOR status to modulate cell cycle tuning in response to glucose signaling.

Taken together, this work revealed *ABL1* as a critical lynchpin connecting: 1) glucose and terminal appressorial cell differentiation, 2) glucose and TOR activity, and 3) glucose and cell cycle regulation.

### Insights on glucose control of TOR

In yeast, AMPK/Snf1 represses downstream TOR pathway branches under low energy conditions in order to activate the glucose-starvation response [45]. Here, we showed conversely that *ABL1* acts upstream of TOR in order to inactivate the glucose-sufficiency response. Although the *ABL1* gene was expressed in response to G6P sensing by Tps1 under glucose-replete conditions, the Abl1 protein was detected under both glucose-limiting and glucose-sufficient growth conditions. However, our 2-DOG results demonstrated that glucose or G6P (rather than ATP/AMP levels) inhibited Abl1 protein activity in order to activate TOR under glucose-sufficient conditions. Considered together, these results are consistent with Abl1 operating as a fast acting brake on TOR function under glucose-limiting conditions.

Genes encoding AMP/Snf1 kinases and their associated β- and γ- subunits are found in the genomes of many fungi, including *M*. *oryzae* [32]. However, Abl1 is not likely an AMPK β- subunit because the Δ*abl1* phenotype does not resemble that of the *M*. *oryzae* AMPK β- subunit deletion strain Δ*mosip2*, which forms appressoria [32], and Abl1 lacks the iteration domain of AMPK β- subunits. Moreover, the Abl1 protein sequence aligns most closely in yeast (39% identity) with MDG1/YNL173C [46], a little characterized membrane bound *S*. *cerevisiae* protein that also carries the AMPK β-like GBD domain but not the AMPK β-subunit iteration domain and is involved in a complex genetic pathway linking pheromone signalling and cell polarity. Interestingly, MDG1/YNL173C physically interacts with ATG1 on a proteome chip, although the mechanism and physiological significance is not known [47]. ATG1 is a downstream substrate of TOR, but evidence from *Drosophila* suggests ATG1 can also be an upstream regulator of TOR [48]. MDG1/YNL173C is also a component of the yeast eisosome [49], a membrane microdomain that interacts with TORC2 [50]. Although Abl1, according to PSORTII, is not predicted to be membrane localized, these potential links between MDG1/YNL173C and TOR in yeast might give tantalizing hints about how Abl1, and thus glucose, regulates TOR in *M*. *oryzae*. Exploring the biochemical connections between glucose/G6P, Abl1 and TOR will be a future goal.

### Insights on glucose control of the cell cycle

A surprising outcome of this work has been deducing the role of glucose in *M*. *oryzae* cell cycle regulation. In contrast to genotoxic stresses, the molecular mechanisms underlying glucose cell cycle checkpoints are not well understood [51]. Here, we showed how *ABL1* regulates TOR and tunes the cell cycle in order to determine cell morphogenesis in response to glucose or G6P (Fig 9). Interestingly, the effect of glucose treatment or *ABL1* loss on cell cycle progression was stochastic such that, in contrast to Fig 9A which holds true for all germinating WT spores under glucose starvation conditions, the scheme in Fig 9B is inherently unstable: some germinating Δ*abl1* or glucose treated WT spores undertook more rounds of mitosis and septation than others while 25% of such spores formed morphologically normal (Type IV) appressoria with single nuclei and one septation event. Moreover, multiple rounds of septation and misregulated (rather than delayed) autophagy and glycogen mobilization were cell type-specific defects associated with Type I-III Δ*abl1* morphotypes rather than resulting directly from the loss of *ABL1*. We thus propose that glucose treatment or the loss of *ABL1* increases cell cycle variability such that cell cycle sharpness is lost and appressorium development becomes stochastic rather than deterministic. This stochasticity or loss of coherence is reminiscent of sporadic unbudded arrest in *cln2*Δ *cln1*Δ double mutants of yeast, where 26% of cells failed to bud due to the loss of a positive feedback loop ensuring robust cell cycle entry [52]. Similarly, *ABL1* might be required to eliminate stochastic variability and boost the robustness of cell cycle regulation by providing feedback reinforcement during appressorial differentiation. This could be achieved using a bistable switch that toggles between two discrete states (ie. TOR_off_ and TOR_on_, Fig 9A) and prevents cell cycle slippage by promoting settling into mutually exclusive interphase and M-phase states [53]. Hysteresis (memory) is a defining feature of a bistable switch [53]. The commitment of appressorial nuclei to G0 following G2 arrest, even if glucose is added between these two phases, indicates hysteresis because one state (TOR_off_ at G0) is dependent on a previous state (TOR_off_ at G2 delay). Thus, *ABL1* might create a bistable switch to enforce robust cell cycle entry under glucose starvation conditions. Conversely, glucose or glucose mimicking Δ*abl1* mutants would perturb the putative bistable switch (and hysteresis) by facilitating a constitutive TOR_on_ status resulting in the stochastic loss of cell cycle robustness. Treatments that increase cell cycle variability could thus be leveraged as a means to eliminate appressorium formation by important phytopathogens.

### Conclusion

This study unveiled a novel glucose-mediated signaling axis and showed it engaged metabolic checkpoints at G2/M and G1/G0 in order to modulate cell cycle tuning and control appressorium development. Mechanistically, Abl1 negatively regulated TOR function in response to the absence of glucose or G6P, demonstrating a novel connection between cellular glucose and TOR activity that sheds light on how TOR receives inhibitory signals. We also demonstrated direct connections between glucose and cell cycle regulation by elucidating how TOR can tune mitosis in response to the presence or absence of cellular glucose. Collectively, this study demonstrates the utility of using *M*. *oryzae* to enhance our understanding of the factors controlling cell differentiation. We suggest that this relatively simple system could be leveraged towards further fundamental discoveries in development that might impact our understanding of the pathologies resulting from TOR and/or cell cycle dysregulation. This might be achieved by exploiting, for example, appressorial differentiation as an adjustable TOR readout.

## Materials and Methods

### Strain maintenance and pathogenicity tests

Strains were grown at 24°C on complete medium (CM) or Cove's minimal nitrate media (MM) [28,54], unless otherwise noted. WT and mutant strains used in this study are listed in S4 Table. Fungal spores were isolated from 12–14 day-old plate cultures and resuspended at a rate of 1×10^5^ conidiospores/ ml in 0.2% gelatin. Three-week-old seedlings of susceptible rice (*Oryza sativa*) cultivar, CO-39, were used for spray assays as described previously [28]. Lesion formation was examined 5-day post-inoculation. Images of the infected leaves were taken by using an Epson Perfection V550 scanner at a resolution of 600 dpi.

Live-cell imaging was performed at 22°C as described previously [55] using 3 cm-long leaf sheath segments from three week-old rice plants and injecting one end of the sheath with a spore suspension of 1 x 10^5^ spores/ml in 0.2% gelatin. At the time points indicated, leaf sheaths were trimmed and observed using a Nikon Eclipse 50i microscope and a Nikon D100 digital net camera. The average rates of appressorium formation and penetration, and IH cell-to-cell movement from the first infected cell, were determined for each strain, in triplicate, by analyzing 50 spores or appressoria per rice cuticle [55].

### Targeted gene replacement and complementation

All targeted gene deletion mutants were generated using the split marker approach described by Wilson et al [26], in which a selectable marker replaces all or part of the native gene of interest. The *ABL1* gene was replaced in Guy11 (WT), Guy11 H1:RFP (WT H1:RFP), Δ*tps1*, and the Δ*rbp35* parental backgrounds using the *ILV1* gene conferring resistance to sulphonyl urea [26]. Primers were designed to amplify a 1 kb sequence upstream and a 1 kb sequence downstream of *ABL1* (S5 Table). The *Δabl1* mutant strain was complemented with the full length *ABL1* gene, or *ABL1* fused to GFP, using plasmids that were constructed by the yeast GAP-repair approach described in Li et al. [54], and the primers listed in S5 Table. Constructs were transformed into *Δabl1* protoplasts and transformants were selected by hygromycin resistance [19,26].

### Transcript analysis

WT and *Δtps1* strains were grown for 48 h in CM before switching to 1% glucose minimal media, or glucose starvation media, as indicated, for 16 h. Mycelia were frozen in liquid nitrogen, and lyophilized for 36 hrs. RNA was extracted from fungal mycelium using the RNeasy mini kit from Qiagen. RNA was converted to cDNA using the qScript reagents from Quantas. Real time quantitative PCR was performed on an Eppendorf Mastercycler Realplex using the recommended reagents with the specific primers for *ABL1* and *TUB1* showed in S5 Table. qPCR data was analyzed using the Realplex software and the ΔΔCt method [56]. Values are the average of three results from at least two independent biological replicates. Thermocycler conditions were: 10 min at 95°C, followed by 40 cycles of 95°C for 30 sec, 63°C for 30 sec and 72°C for 30 sec.

### Appressorial development and cell cycle analysis

Fungal spores were collected from 12–14 day-old plate cultures and resuspended at a rate of 1×10^5^ conidia/ml. 200 μl of the spore suspensions were inoculated onto hydrophobic plastic coverslips (Fisherbrand) and/or hydrophilic glass slides (Fisherbrand) to evaluate appressoria formation at 24 hpi. All treatments were performed at 22°C. Appressorium formation rates were determined by counting the number of appressoria formed from 50 spores per coverslip or slide, repeated in triplicate for each strain and treatment [19]. Nuclei number was determined from 100 spores per coverslip, repeated in triplicate for each strain and treatment. The following treatments at the respective final concentrations were added to the spore suspensions at the indicated time points and analyzed at 24 hpi: 100–200 nM Rapamycin (Rap; LC Laboratories, USA), 10 mM monobutyryl cyclic AMP (cAMP; Sigma-Aldrich, USA), 50 mM hydroxyurea (HU; Fisher Scientific, USA), 30 μM benomyl (Ben; Fisher Scientific, USA), 2 mM cyclohexamide (CHX; Sigma-Aldrich, USA), 1% (w/v) glucose (Glc; Fisher Scientific, USA), 5 mM autophagy inhibitor 3-MA (Fisher Scientific, USA), 0.02% (w/v) calcofluor white (Sigma Aldrich, USA), 100 mM Monodansylcadaverine Crystallin (MDC; Sigma-Aldrich, USA), 0.5% (w/v) 2-deoxyglucose (2-DOG; Fisher Scientific, USA) and 5 mM L-glutamine (Fisher Scientific, USA). Images were taken using a Nikon A1 laser scanning confocal mounted on a Nikon 90i compound microscope (software version: NIS Elements 4.13 Build914) at the University of Nebraska-Lincoln Morrison Microscopy Core Research Facility. Transmitted light and fluorescence for td tomato were imaged with a 561.5 nm laser. td tomato fluoresce was detected at 570–620 nm. MDC and calcofluor white fluorescence was detected at 425–475 nm.

## Supporting Information

S1 Fig *ABL1* is not required for carbon metabolism. (A) *ABL1* gene expression was analyzed in WT strains following 16 hours of growth in 1% (w/v) glucose minimal media (GMM) with nitrate as the sole nitrogen source (+N) or without a nitrogen source (-N). Transcript abundance was normalized against *β*-tubulin (*TUB2*) gene expression for each condition. (B) The *Δabl1* mutant strain was not impaired in spore production compared to WT. Spores were harvested from plates of GMM with nitrate as the nitrogen source following 12 days of growth. (C) The *Δabl1* mutant strain was not defective in glucose uptake. WT and *Δabl1* strains were grown for 10 days on 85 mm petri-dishes containing minimal media with glucose as the sole carbon source at the final concentrations (w/v) shown, and with 10 mM nitrate (NO_3_^-^) as the sole nitrogen source. (D) Strains were grown for 10 days on minimal media supplemented with the indicated carbon sources and 10 mM NO_3_^-^ as the sole nitrogen source. Deleting the *ABL1* gene did not affect growth compared to WT. (A, B) Values are the mean of at least three independent replicates. Error bars are standard deviation. NS: not significantly different (*Student’s t-test p* ≥ 0.05).
(JPG)

S2 Fig *ABL1* is downstream of *TPS1*. (A) The *Δabl1Δtps1* double mutant strain, but not the *Δabl1* single mutant, was nitrate non-utilizing, suggesting *TPS1* is epistatic to *ABL1*. Strains were grown on 85 mm petri-dishes containing complete media (CM) and 1% (W/V) glucose minimal media (GMM) with 10 mM nitrate (NO_3_^-^) or ammonium (NH_4_^+^) as the sole nitrogen source. Images were taken 10 days after inoculation. (B) *TPS1* transcript abundance relative to *β*-tubulin (*TUB2)* expression was not significant different (NS, *Student’s t-test p* ≥ 0.05) between WT and *Δabl1* mutant strains, following growth in liquid GMM with 10 mM NO_3_^-^ as the sole nitrogen source. (C) The average rate of appressorium formation on the rice leaf surface at 24 hpi was determined for WT and *Δtps1* strains. Spore suspensions (5 x 10^4^ spores /mL) of each strain were inoculated onto rice cuticles. Rapamycin (Rap) was added at the indicated concentration. Rates were quantified from 50 conidia of each strain with and without Rap. (D) Appressorial penetration rates were calculated from a total of 50 appressoria observed for each strain. (C-D) Values are the mean of six independent replicates. Error bars are standard deviation. NS: not significantly different (*Student’s t-test p* ≥ 0.05) (E) Treating spores with rapamycin did not restore *Δtps1* growth in rice cells. At 44 hpi, *Δtps1* IH are restricted to the first penetrated cell, regardless of rapamycin treatment. Black arrows indicate the penetration site and scale bars are 5 μm. (C-E) NT = no treatment control.
(JPG)

S3 Fig *ABL1* is required for appressorium formation and function and for *M*. *oryzae* growth *in planta*. Appressorium formation rates on (A) artificial hydrophobic surfaces and (B) rice leaf surfaces were significantly reduced (*Student’s t-test* p ≤ 0.05) in *Δabl1* mutant strains compared to WT and the *Δabl1* complementation strain. Appressorium formation rates were calculated from 50 spores per coverslip or leaf sheath, repeated in triplicate, at 24 hpi. (C) Less than 20% of *Δabl1* appressoria could penetrate rice cuticles into the underlying epidermal cells. Appressorium penetration rates were calculated from 50 appressoria per leaf sheath, repeated in triplicate, at 24 hpi. (D) For those *Δabl1* appressoria that did penetrate into underlying epidermal cells, *Δabl1* IH development and movement to cells adjacent to the penetration point (*black arrows*) was significantly (*Student’s t-test* p ≤ 0.05) impaired compared to WT and the *Δabl1* complementation strain at 44 hpi. Asterisks indicate IH movement from primary infected cells to neighboring cells. Scale bars are 5 μm. (E) Calculated rates of IH movement to adjacent cells per 50 penetrating appressorium, per strain, repeated in triplicate. (A-C, D) Values are the mean of three independent replicates. Error bars are the standard deviation. Bars with different letters are significantly different (*Student’s t-test* p ≤ 0.05). (A-E) Coverslips and detached rice leaf sheaths were inoculated with spore suspensions at a rate of 1 x 10^5^ spores/mL.
(JPG)

S4 Fig The *Δabl1* H1:RFP mutant strain derived from Guy11 H1:RFP resembles the *Δabl1* mutant strain derived from Guy11. (A) Five days after inoculation, the *Δabl1* H1: RFP strain was non-pathogenic on susceptible CO-39 rice seedlings. (B) Germinating Δ*abl1* H1:RFP spores displayed four morphotypes, and responded to Rapamycin (Rap) but not cAMP treatment, on hydrophobic surfaces at 24 hpi. Type I-IV morphotypes are designated according to Fig 1C. (C) At 44 hpi on detached rice leaf sheaths, *Δabl1* H1:RFP mutant strains were impaired in cell-to-cell movement compared to WT H1:RFP. Black arrows show appressoria on the leaf surface and asterisks indicates IH movement from primary infected cells to neighboring cells. Scale bars are 5 μm. Merged DIC and fluorescence images are shown.
(JPG)

S5 Fig Glycogen mobilization was perturbed in Type I-III morphotypes of Δ*abl1* mutant strains. Glycogen mobilization was impaired in *Δabl1* mutant morphotypes compared to WT. Asterisks indicate glycogen accumulation and show that, consistent with previous reports, by 10 hpi, glycogen had mobilized from WT spores into incipient appressoria, where it was fully degraded by 24 hpi. In contrast, glycogen was still present in the spores of Type I-III Δ*abl1* morphotypes by 10 hpi. By 24 hpi, glycogen was concentrated in the spores of Type I and III Δ*abl1* morphotypes and was concentrated in the appressoria of Type II Δ*abl1* morphotypes. Glycogen mobilization in Type IV morphotypes was similar to WT but glycogen degradation in some appressoria was delayed. Glycogen was stained by the KI and I_2_ mixture for five minutes at the indicated time points.
(JPG)

S6 Fig Glucose signals through TOR via *ABL1*. (A) 0.02% (w/v) calcafluor white staining shows septation is misregulated in Type I-III Δ*abl1* morphotypes. (B) Treatment with 1% (w/v) glucose (Glc) reduced appressoria formation in WT and the rapamycin insensitive Δ*fpr1* mutant strain. Adding 200 nM rapamycin (Rap) to glucose treated spores induced appressorium formation in WT but not Δ*fpr1* mutant strains. Appressorial formation rates were determined at 24 hpi from 50 spores per hydrophobic coverslip, repeated in triplicate. NT = no treatment. Glc is 1% (w/v), Rap is 200 nM rapamycin. Error bars are the standard deviation. Bars with different letters are significantly different (P ≤ 0.05). (C) Proportion of WT H1:RFP and Δ*abl1* H1:RFP strains carrying 1, 4, 5 or 6 nuclei at 24 hpi. NT = no treatment control. Glc = spores treated with 1% (w/v) glucose. Nuclei number was determined for 100 germinating conidia per coverslip, repeated in triplicate for each strain and treatment. Error bars are standard deviation. Bars with different letters are significantly different (P ≤ 0.05). (D) The number of nuclei carried by germinating WT H1:RFP spores at the indicated time points, at 22°C, following treatment with 1% Glc (w/v) at 0 hpi. Mean values were calculated from three independent replicates by counting the nuclei from 100 spores per time point per strain for each replicate. Error bars are standard deviation.
(JPG)

S7 Fig Abl1 controls mitosis under glucose-limiting conditions. Strains were grown in liquid CM for 48 h and harvested. An equal weight of wet mycelia (20 g) of each strain was inoculated into 100 mL of fresh liquid CM lacking glucose (-Glc). Mycelia was harvested from this media at 2 hpi and analyzed. (A) Abl1^GFP^ was detected following growth in the presence and absence of glucose by Western Blot analysis. Strains of Δ*abl1 Abl1*^*GFP*^ were grown in CM for 48 h then transferred to CM with (+) and without (-) 1% (w/v) Glc as a carbon source for 2 h. Total protein extracts were obtained by grinding 200 mg of fungal mycelium in liquid nitrogen and re-suspending in 400 μl of 2X sample buffer (100 mM Tris-HCl pH 6.8, 4% (w/v) SDS, 0.2% (w/v) bromophenol blue, 20% (v/v) glycerol, 200 mM DTT, 5% (v/v) β-mercaptoethanol). Samples were incubated for 5 minutes at 95°C and then centrifuged at 4,700 rpm for 5 min. Protein samples (30 μl) from each extract were fractionated by SDS-PAGE, transferred to Immun-Blot^®^ PVDF membrane (Bio-Rad, USA) and immunoblotted with monoclonal α- GFP (1:10000 dilution; Sigma-Aldrich, USA) and α-Tubulin (1: 10000 dilution; Santa Cruz Biotechnology, USA). Secondary antibodies were used at 1: 10000 dilutions. The Clarity Western ECL substrate (Bio-Rad, USA) was used to develop the blots. Images were taken with the ChemiDoc XRS+ (Bio-Rad, USA), using the Chemi Hi Resolution application. (B) The bands in (A) were analyzed using Image Lab (software version 5.2.1, Bio-Rad). Relative GFP signal intensity was obtained by normalizing against α-Tubulin and correcting for the background determined from a WT control strain. (C) The number of nuclei per 100 0μm of hyphae was calculated using ImageJ software (rsbweb.nih.gov/ij). (D) The Δ*abl1* H1:RFP mutant strain carried more nuclei than WT after growth in CM -Glc. Scale bar is 20 μm. (E) The Δ*abl1* H1:RFP mutant strain had more mass than WT after growth under glucose starvation conditions. After 2 h of growth in the media lacking Glc, samples were harvested and lyophilized for 36 h. The dry weight of the mycelia was quantified for each strain. (C, E) Results are the mean of three independent replicates. Values are statistically significant at *P < 0.05 (*Student’s t-test*). Error bars are standard deviation.
(JPG)

S8 Fig Resolving cell cycle phase boundaries. (A) WT H1:RFP spores treated with 30 μM Ben at 6hpi form aberrant appressoria and complete nuclear degeneration indicating G2 arrest occurs after 6 hpi. (B) WT H1:RFP and Δ*abl1* H1:RFP spores treated with 50 mM cycloheximide at 2 hpi and 3 hpi. (A,B) Merged DIC and fluorescence image was obtained at 24 hpi. Scale bars are 10 μm.
(JPG)

S9 Fig Glutamine treatment of WT spores partially abolishes appressorium formation. (A) Germinating spores of WT treated, or not treated (NT), with 5 mM glutamine were viewed at 24 hpi. Treatment induced long germ tubes and prevented appressorium formation by some germinating spores. Scale bars are 10 μm. (B) By 24 hpi, compared to NT controls, glutamine treated WT spores displayed the Type I, II and IV morphotypes observed for germinating Δ*abl1* and glucose treated WT spores (Fig 6C), but not in the same proportions, and absent Type III, suggesting glutamine and glucose might activate TOR by different pathways. The large proportion of treated germinating spores displaying Type IV morphotype by 24 hpi might reflect reduced uptake of glutamine due to its poor solubility in water. Type I-IV morphotypes are designated according to Fig 1C. Gln = 5 mM glutamine. NT = no treatment.
(JPG)

S10 Fig The loss of *ASD4* affects autophagy, glycogen mobilization, septation and mitosis during appressorium formation. (A) Monodansylcadaverine (MDC) staining shows autophagosome distribution was affected in Δ*asd4* germinating spores compared to WT by 24 hpi. Type I and III morphotypes were observed for Δ*asd4* mutants and the autophagosome distribution displayed the same patterns as those of Δ*abl1*. Scale bars are 10 μm. (B) Glycogen was stained by KI and I_2_ mixture for five minutes at the indicated time points. Arrows indicate the aggregation of glycogen in both Δ*asd4* and WT germinating spores. By 10 hpi, glycogen had mobilized from WT spores into incipient appressoria, where it was fully degraded by 24 hpi. In contrast, glycogen was still present in the conidial area in both morphotypes of Δ*asd4* spores by10 hpi and 24 hpi. Stars indicate glycogen accumulation. (C) Δ*asd4* germinating spores presenting as Type I and III morphotypes displayed more septation events in germ tubes than WT (Fig 6B). Cell walls were stained by calcofluor white and 10% KOH at 24 hpi. Scale bars are 10 μm. (D) Nuclei were visualized by the SYTO green fluorescent nucleic acid stain. 200 μl spore suspension at 1X10^4^ spores/ml, was inoculated onto hydrophobic plastic coverslip and incubated in dark for 24 hours at room temperature. 10 μl of 1:1000 SYTO green (Fisher, USA) solution was added to the spore suspension and gently mixed on the plastic coverslip. Images were taken 30 mins after SYTO green staining by confocal microscopy. The samples were excited with the 488 nm filter and viewed with the 525 nm laser. Scale bar is 10 μm.
(JPG)

S1 Table Morphotypes formed by germinating conidia at 24 hours post inoculation on hydrophobic surfaces. (DOCX)

S2 Table Number of nuclei carried by germinating conidia at 24 hours post inoculation on artificial hydrophobic surfaces at 22°C. (DOCX)

S3 Table Percentage of appressoria formed at 24 hpi by germinating WT and *Δabl1* spores at 22°C. (DOCX)

S4 Table *Magnaporthe oryzae* strains used in this study. (DOCX)

S5 Table Oligonucleotide primers used in this study. (DOCX)
