# Supplementary figures and images for: Glucose-ABL1-TOR Signaling Modulates Cell Cycle Tuning to Control Terminal Appressorial Cell Differentiation
Source: PLoS Genet. 2017 Jan 10;13(1):e1006557. doi: 10.1371/journal.pgen.1006557 (PMC5266329; doi:10.1371/journal.pgen.1006557)

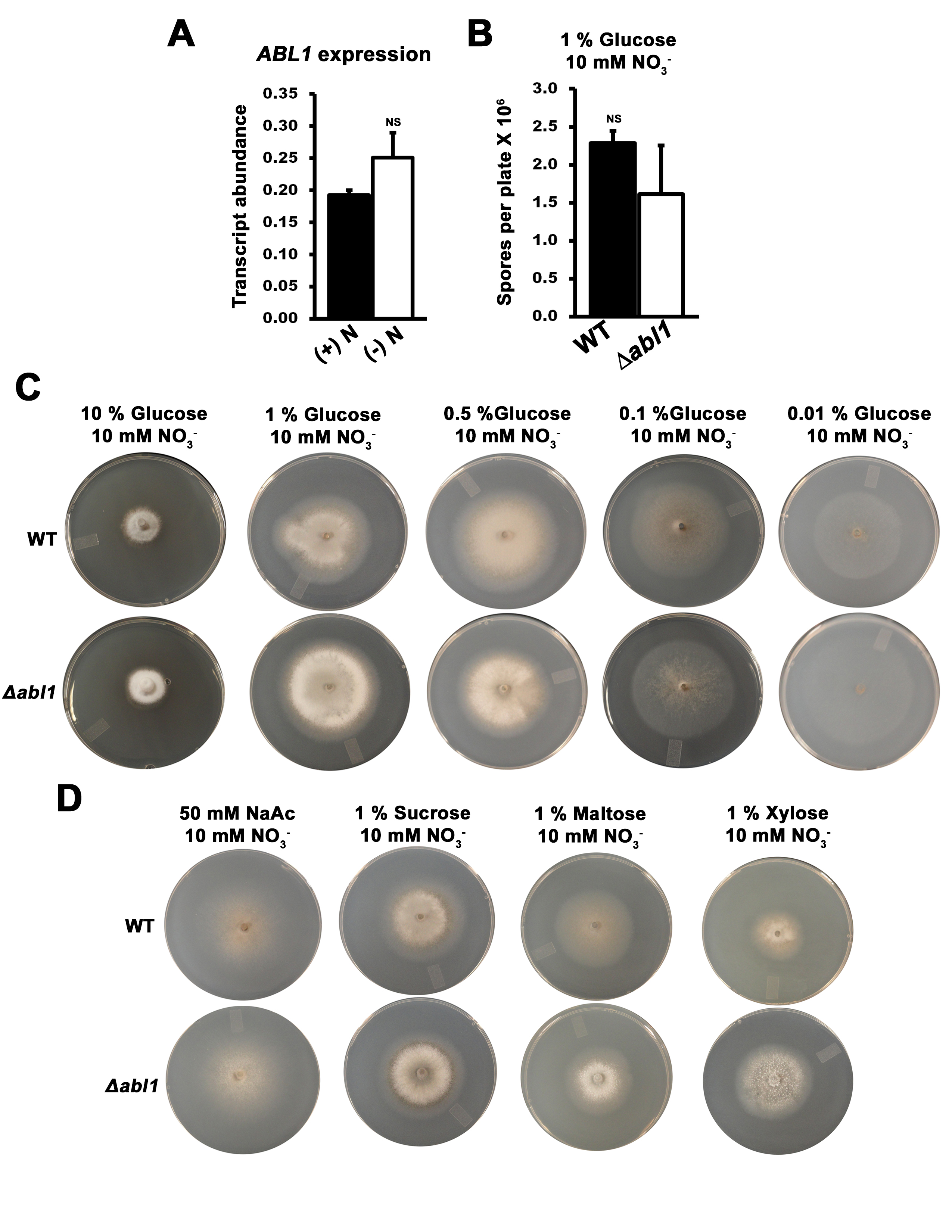

Supplement: S1 Fig — (A) ABL1 gene expression was analyzed in WT strains following 16 hours of growth in 1% (w/v) glucose minimal media (GMM) with nitrate as the sole nitrogen source (+N) or without a nitrogen source (-N). Transcript abundance was normalized against β-tubulin (TUB2) gene expression for each condition. (B) The Δabl1 mutant strain was not impaired in spore production compared to WT. Spores were harvested from plates of GMM with nitrate as the nitrogen source following 12 days of growth. (C) The Δabl1 mutant strain was not defective in glucose uptake. WT and Δabl1 strains were grown for 10 days on 85 mm petri-dishes containing minimal media with glucose as the sole carbon source at the final concentrations (w/v) shown, and with 10 mM nitrate (NO3-) as the sole nitrogen source. (D) Strains were grown for 10 days on minimal media supplemented with the indicated carbon sources and 10 mM NO3- as the sole nitrogen source. Deleting the ABL1 gene did not affect growth compared to WT. (A, B) Values are the mean of at least three independent replicates. Error bars are standard deviation. NS: not significantly different (Student’s t-test p ≥ 0.05). (JPG) [file pgen.1006557.s001.jpg]

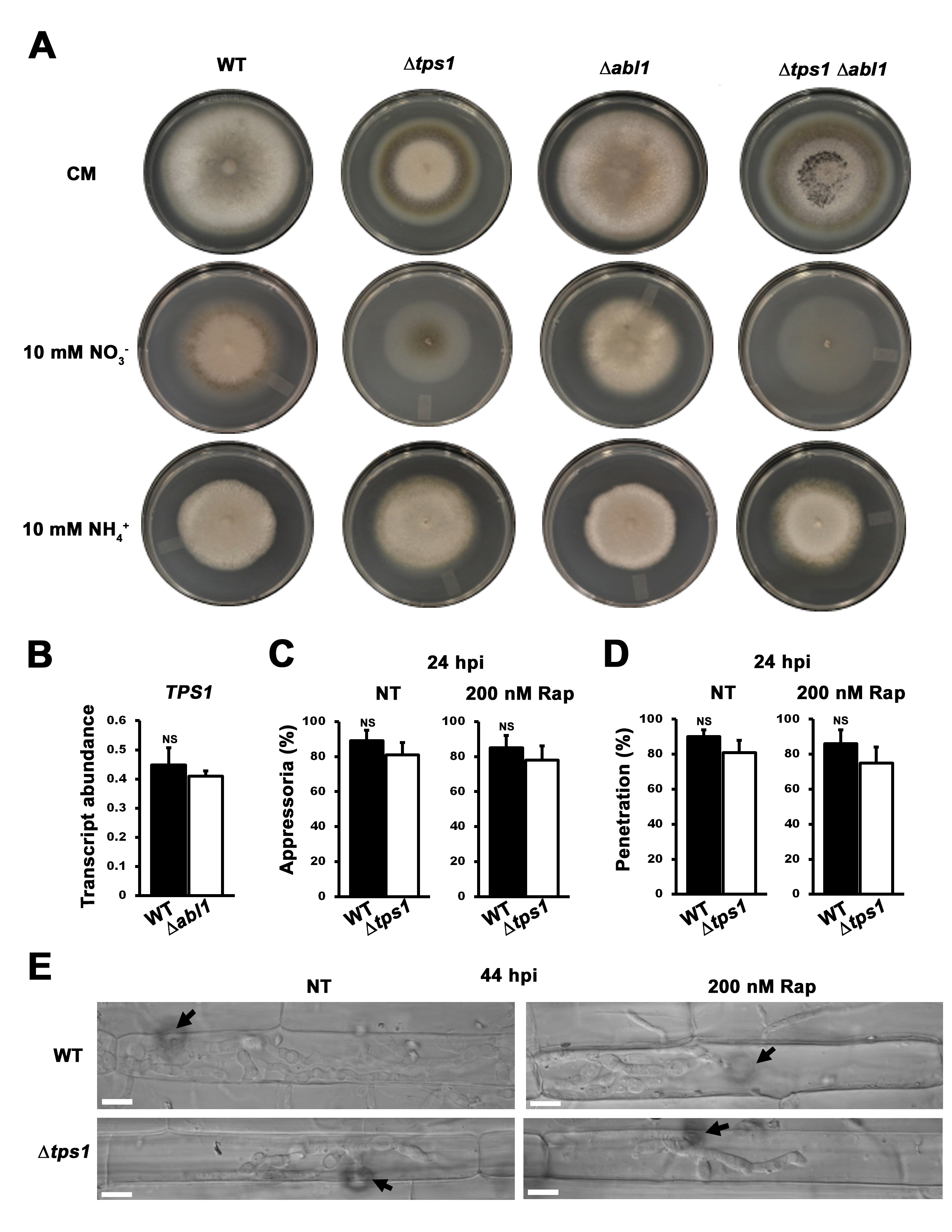

Supplement: S2 Fig — (A) The Δabl1Δtps1 double mutant strain, but not the Δabl1 single mutant, was nitrate non-utilizing, suggesting TPS1 is epistatic to ABL1. Strains were grown on 85 mm petri-dishes containing complete media (CM) and 1% (W/V) glucose minimal media (GMM) with 10 mM nitrate (NO3-) or ammonium (NH4+) as the sole nitrogen source. Images were taken 10 days after inoculation. (B) TPS1 transcript abundance relative to β-tubulin (TUB2) expression was not significant different (NS, Student’s t-test p ≥ 0.05) between WT and Δabl1 mutant strains, following growth in liquid GMM with 10 mM NO3- as the sole nitrogen source. (C) The average rate of appressorium formation on the rice leaf surface at 24 hpi was determined for WT and Δtps1 strains. Spore suspensions (5 x 10^4^ spores /mL) of each strain were inoculated onto rice cuticles. Rapamycin (Rap) was added at the indicated concentration. Rates were quantified from 50 conidia of each strain with and without Rap. (D) Appressorial penetration rates were calculated from a total of 50 appressoria observed for each strain. (C-D) Values are the mean of six independent replicates. Error bars are standard deviation. NS: not significantly different (Student’s t-test p ≥ 0.05) (E) Treating spores with rapamycin did not restore Δtps1 growth in rice cells. At 44 hpi, Δtps1 IH are restricted to the first penetrated cell, regardless of rapamycin treatment. Black arrows indicate the penetration site and scale bars are 5 μm. (C-E) NT = no treatment control. (JPG) [file pgen.1006557.s002.jpg]

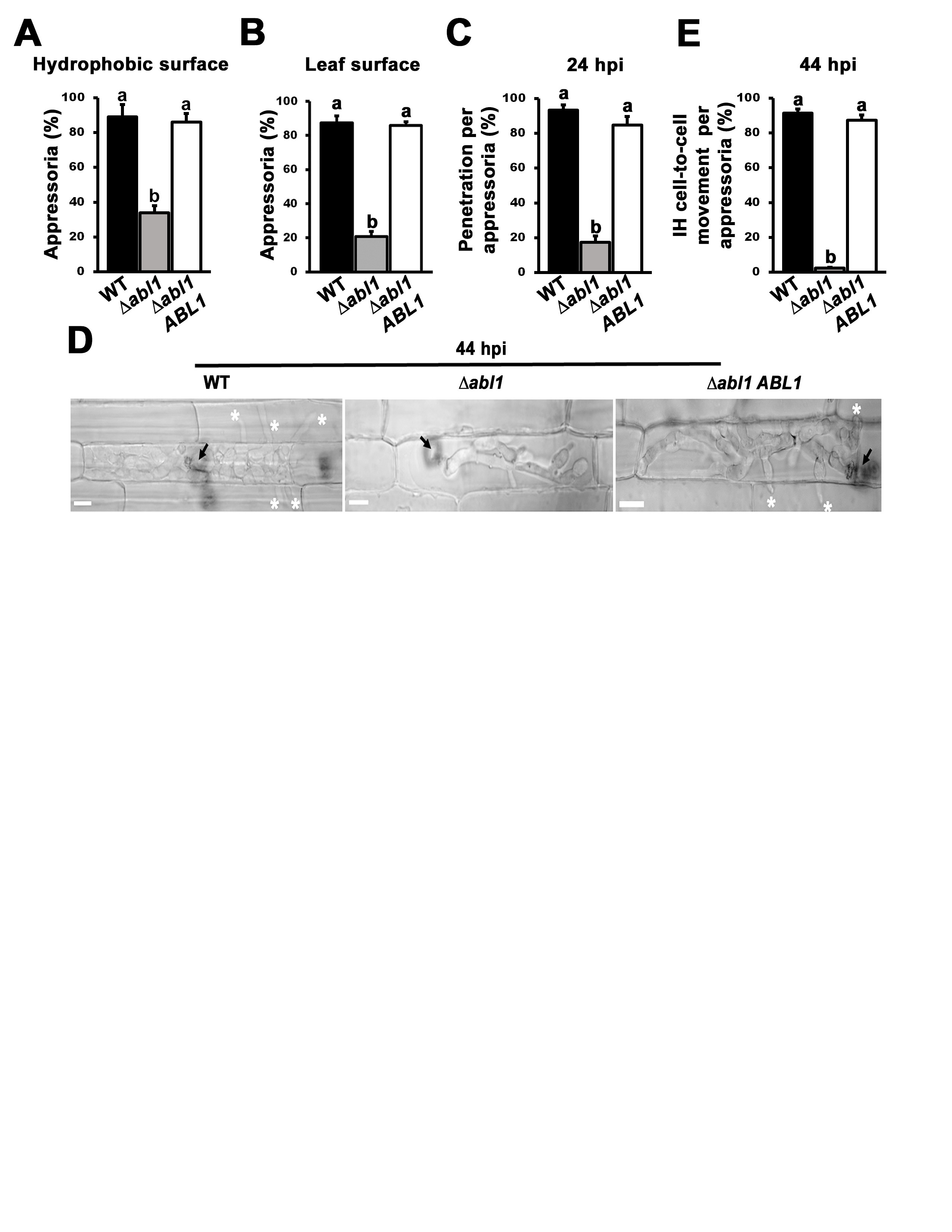

Supplement: S3 Fig — Appressorium formation rates on (A) artificial hydrophobic surfaces and (B) rice leaf surfaces were significantly reduced (Student’s t-test p ≤ 0.05) in Δabl1 mutant strains compared to WT and the Δabl1 complementation strain. Appressorium formation rates were calculated from 50 spores per coverslip or leaf sheath, repeated in triplicate, at 24 hpi. (C) Less than 20% of Δabl1 appressoria could penetrate rice cuticles into the underlying epidermal cells. Appressorium penetration rates were calculated from 50 appressoria per leaf sheath, repeated in triplicate, at 24 hpi. (D) For those Δabl1 appressoria that did penetrate into underlying epidermal cells, Δabl1 IH development and movement to cells adjacent to the penetration point (black arrows) was significantly (Student’s t-test p ≤ 0.05) impaired compared to WT and the Δabl1 complementation strain at 44 hpi. Asterisks indicate IH movement from primary infected cells to neighboring cells. Scale bars are 5 μm. (E) Calculated rates of IH movement to adjacent cells per 50 penetrating appressorium, per strain, repeated in triplicate. (A-C, D) Values are the mean of three independent replicates. Error bars are the standard deviation. Bars with different letters are significantly different (Student’s t-test p ≤ 0.05). (A-E) Coverslips and detached rice leaf sheaths were inoculated with spore suspensions at a rate of 1 x 10^5^ spores/mL. (JPG) [file pgen.1006557.s003.jpg]

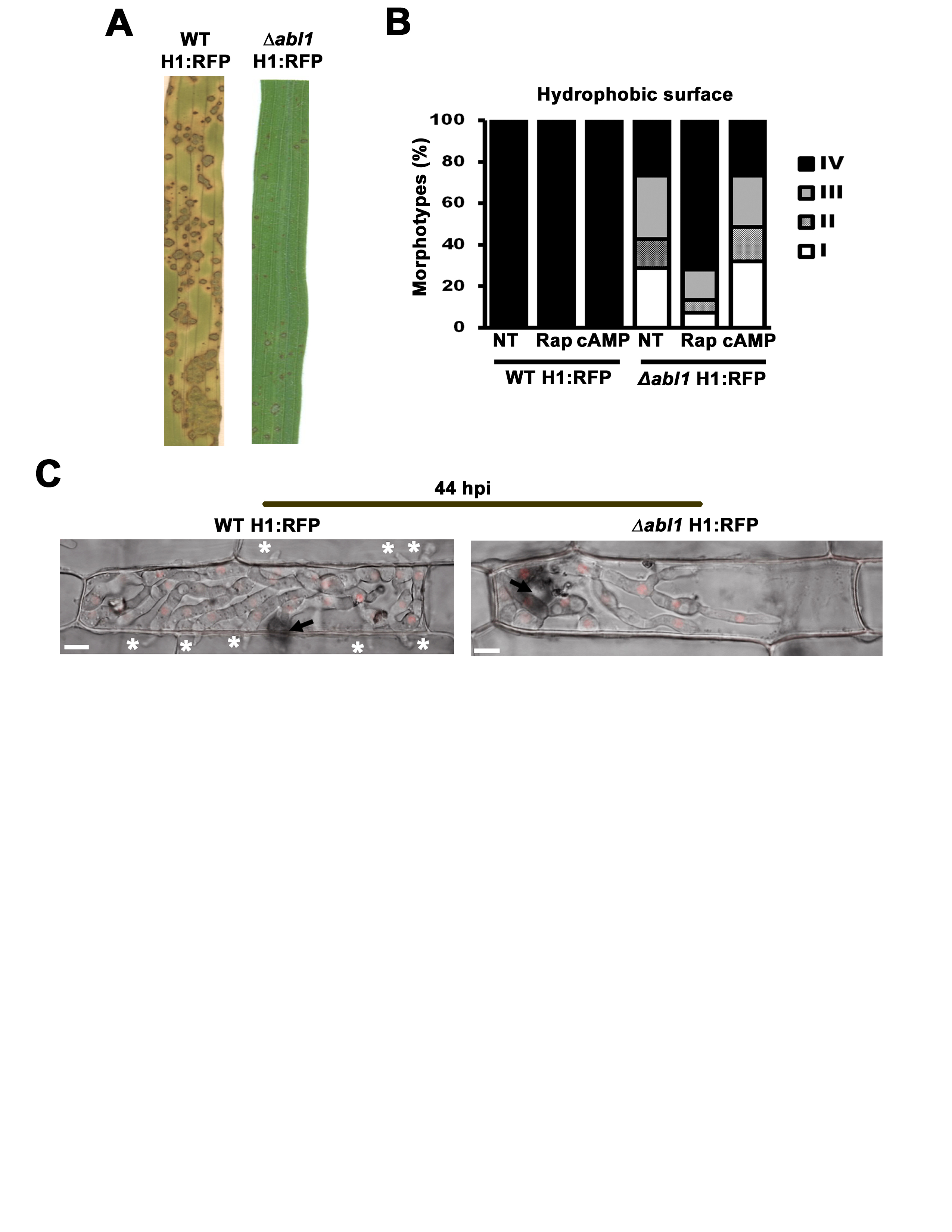

Supplement: S4 Fig — (A) Five days after inoculation, the Δabl1 H1: RFP strain was non-pathogenic on susceptible CO-39 rice seedlings. (B) Germinating Δabl1 H1:RFP spores displayed four morphotypes, and responded to Rapamycin (Rap) but not cAMP treatment, on hydrophobic surfaces at 24 hpi. Type I-IV morphotypes are designated according to Fig 1C. (C) At 44 hpi on detached rice leaf sheaths, Δabl1 H1:RFP mutant strains were impaired in cell-to-cell movement compared to WT H1:RFP. Black arrows show appressoria on the leaf surface and asterisks indicates IH movement from primary infected cells to neighboring cells. Scale bars are 5 μm. Merged DIC and fluorescence images are shown. (JPG) [file pgen.1006557.s004.jpg]

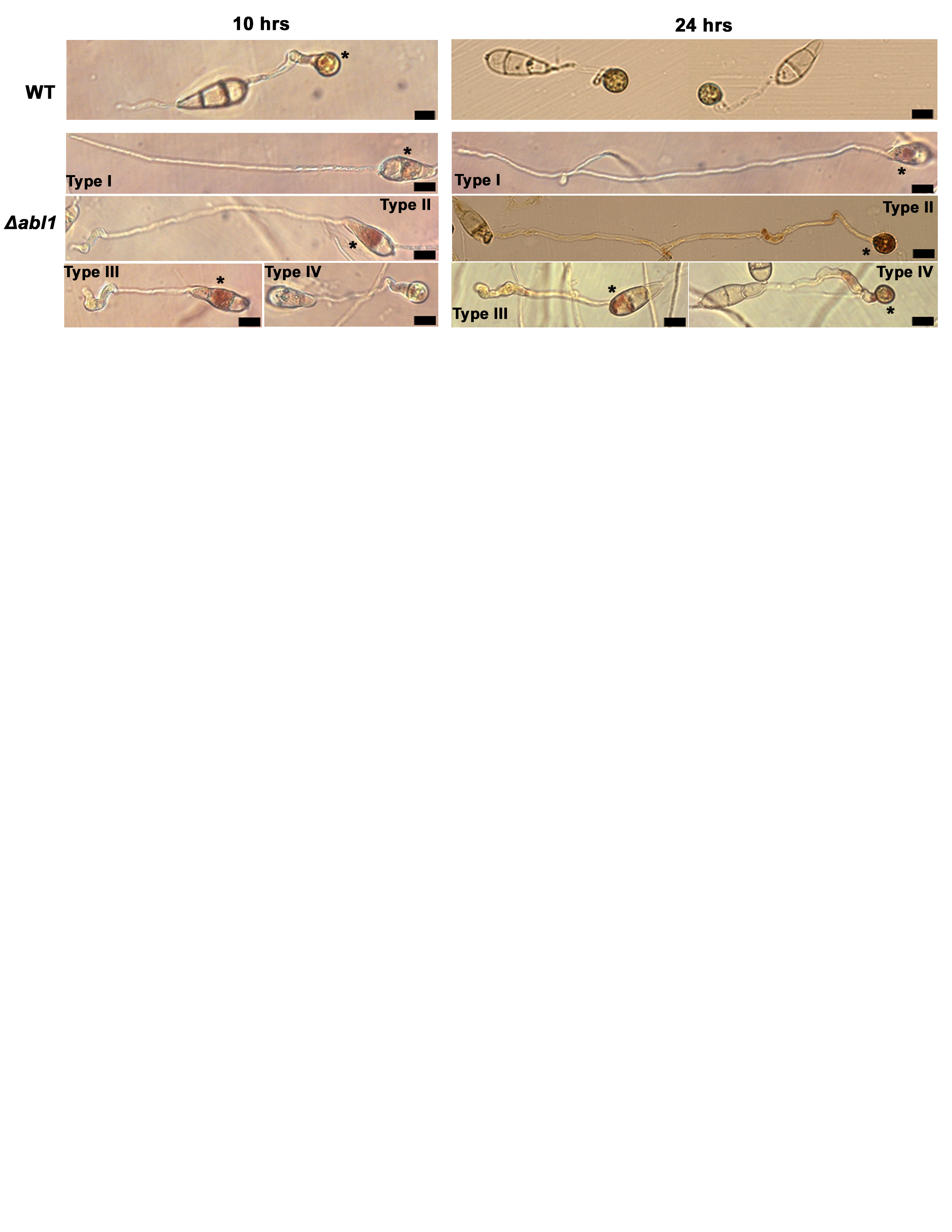

Supplement: S5 Fig — Glycogen mobilization was impaired in Δabl1 mutant morphotypes compared to WT. Asterisks indicate glycogen accumulation and show that, consistent with previous reports, by 10 hpi, glycogen had mobilized from WT spores into incipient appressoria, where it was fully degraded by 24 hpi. In contrast, glycogen was still present in the spores of Type I-III Δabl1 morphotypes by 10 hpi. By 24 hpi, glycogen was concentrated in the spores of Type I and III Δabl1 morphotypes and was concentrated in the appressoria of Type II Δabl1 morphotypes. Glycogen mobilization in Type IV morphotypes was similar to WT but glycogen degradation in some appressoria was delayed. Glycogen was stained by the KI and I2 mixture for five minutes at the indicated time points. (JPG) [file pgen.1006557.s005.jpg]

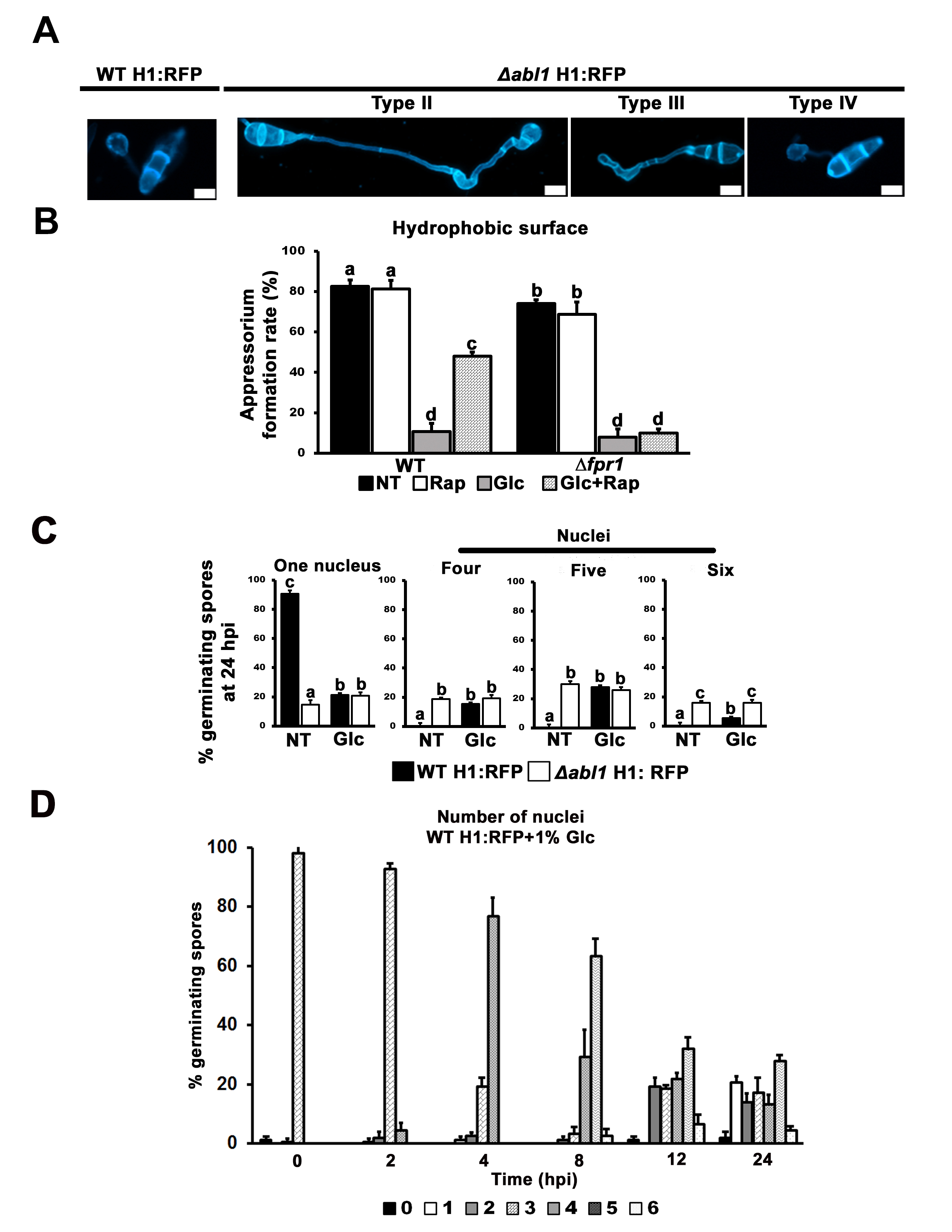

Supplement: S6 Fig — (A) 0.02% (w/v) calcafluor white staining shows septation is misregulated in Type I-III Δabl1 morphotypes. (B) Treatment with 1% (w/v) glucose (Glc) reduced appressoria formation in WT and the rapamycin insensitive Δfpr1 mutant strain. Adding 200 nM rapamycin (Rap) to glucose treated spores induced appressorium formation in WT but not Δfpr1 mutant strains. Appressorial formation rates were determined at 24 hpi from 50 spores per hydrophobic coverslip, repeated in triplicate. NT = no treatment. Glc is 1% (w/v), Rap is 200 nM rapamycin. Error bars are the standard deviation. Bars with different letters are significantly different (P ≤ 0.05). (C) Proportion of WT H1:RFP and Δabl1 H1:RFP strains carrying 1, 4, 5 or 6 nuclei at 24 hpi. NT = no treatment control. Glc = spores treated with 1% (w/v) glucose. Nuclei number was determined for 100 germinating conidia per coverslip, repeated in triplicate for each strain and treatment. Error bars are standard deviation. Bars with different letters are significantly different (P ≤ 0.05). (D) The number of nuclei carried by germinating WT H1:RFP spores at the indicated time points, at 22°C, following treatment with 1% Glc (w/v) at 0 hpi. Mean values were calculated from three independent replicates by counting the nuclei from 100 spores per time point per strain for each replicate. Error bars are standard deviation. (JPG) [file pgen.1006557.s006.jpg]

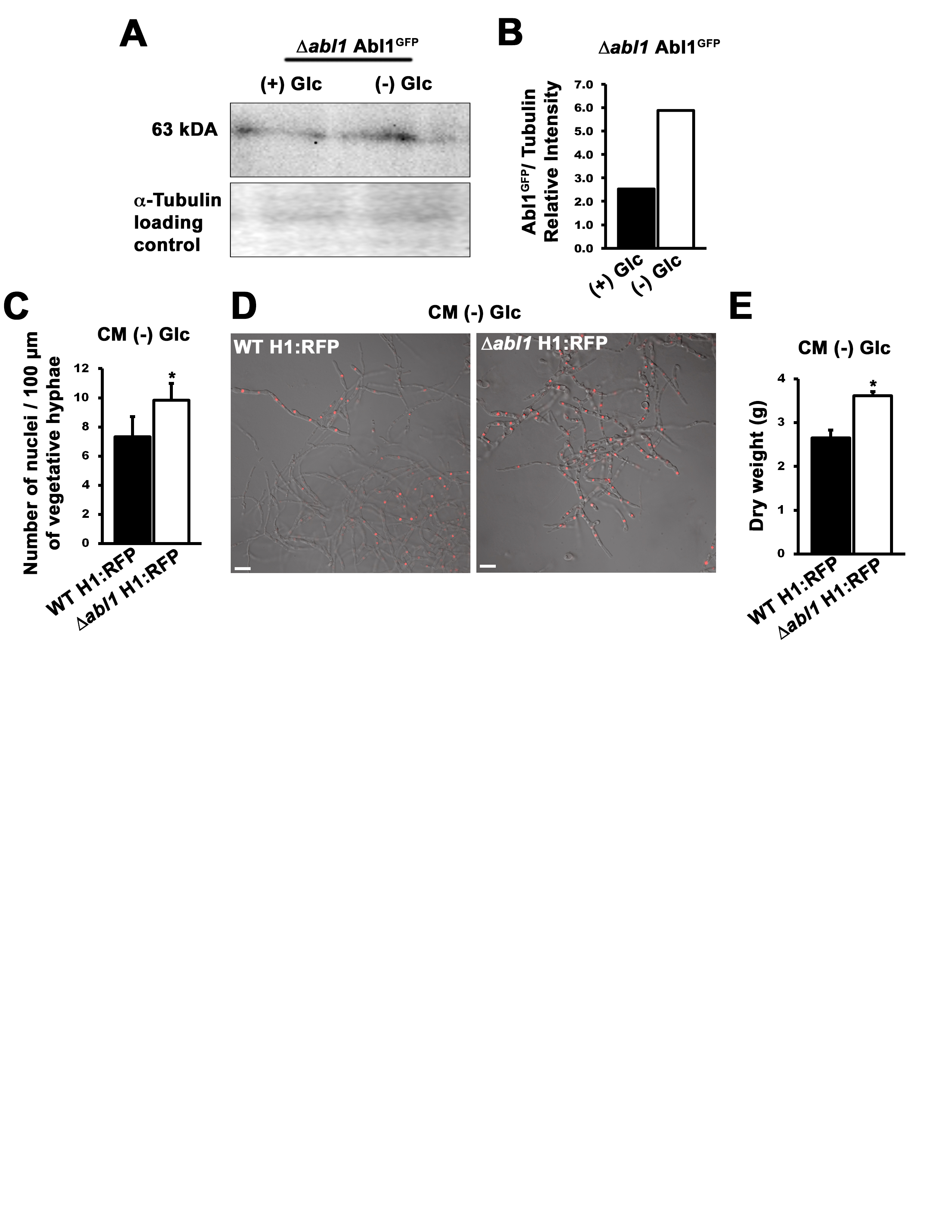

Supplement: S7 Fig — Strains were grown in liquid CM for 48 h and harvested. An equal weight of wet mycelia (20 g) of each strain was inoculated into 100 mL of fresh liquid CM lacking glucose (-Glc). Mycelia was harvested from this media at 2 hpi and analyzed. (A) Abl1GFP was detected following growth in the presence and absence of glucose by Western Blot analysis. Strains of Δabl1 Abl1GFP were grown in CM for 48 h then transferred to CM with (+) and without (-) 1% (w/v) Glc as a carbon source for 2 h. Total protein extracts were obtained by grinding 200 mg of fungal mycelium in liquid nitrogen and re-suspending in 400 μl of 2X sample buffer (100 mM Tris-HCl pH 6.8, 4% (w/v) SDS, 0.2% (w/v) bromophenol blue, 20% (v/v) glycerol, 200 mM DTT, 5% (v/v) β-mercaptoethanol). Samples were incubated for 5 minutes at 95°C and then centrifuged at 4,700 rpm for 5 min. Protein samples (30 μl) from each extract were fractionated by SDS-PAGE, transferred to Immun-Blot® PVDF membrane (Bio-Rad, USA) and immunoblotted with monoclonal α- GFP (1:10000 dilution; Sigma-Aldrich, USA) and α-Tubulin (1: 10000 dilution; Santa Cruz Biotechnology, USA). Secondary antibodies were used at 1: 10000 dilutions. The Clarity Western ECL substrate (Bio-Rad, USA) was used to develop the blots. Images were taken with the ChemiDoc XRS+ (Bio-Rad, USA), using the Chemi Hi Resolution application. (B) The bands in (A) were analyzed using Image Lab (software version 5.2.1, Bio-Rad). Relative GFP signal intensity was obtained by normalizing against α-Tubulin and correcting for the background determined from a WT control strain. (C) The number of nuclei per 100 0μm of hyphae was calculated using ImageJ software (rsbweb.nih.gov/ij). (D) The Δabl1 H1:RFP mutant strain carried more nuclei than WT after growth in CM -Glc. Scale bar is 20 μm. (E) The Δabl1 H1:RFP mutant strain had more mass than WT after growth under glucose starvation conditions. After 2 h of growth in the media lacking Glc, samples were harvested and lyophilized for 3 [file pgen.1006557.s007.jpg]

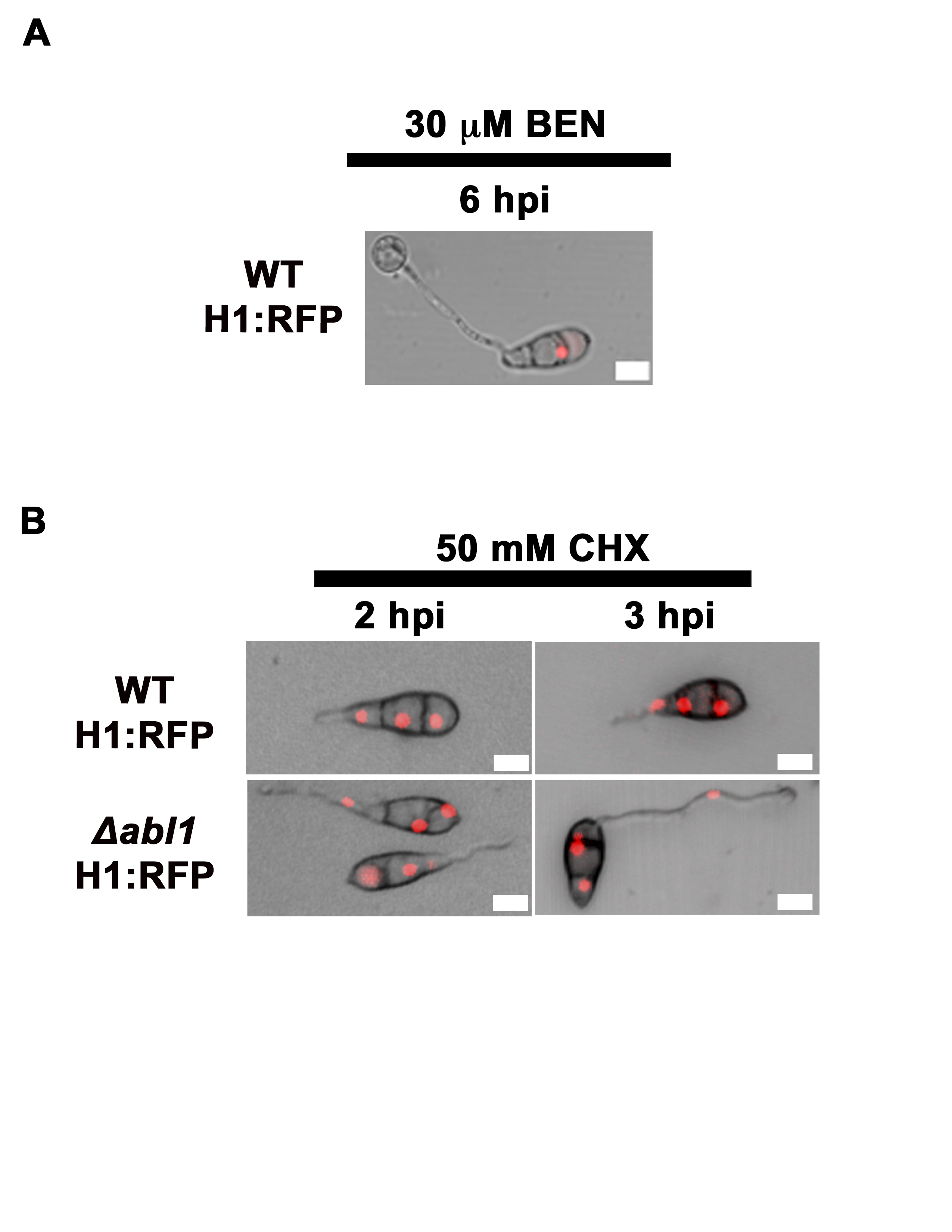

Supplement: S8 Fig — (A) WT H1:RFP spores treated with 30 μM Ben at 6hpi form aberrant appressoria and complete nuclear degeneration indicating G2 arrest occurs after 6 hpi. (B) WT H1:RFP and Δabl1 H1:RFP spores treated with 50 mM cycloheximide at 2 hpi and 3 hpi. (A,B) Merged DIC and fluorescence image was obtained at 24 hpi. Scale bars are 10 μm. (JPG) [file pgen.1006557.s008.jpg]

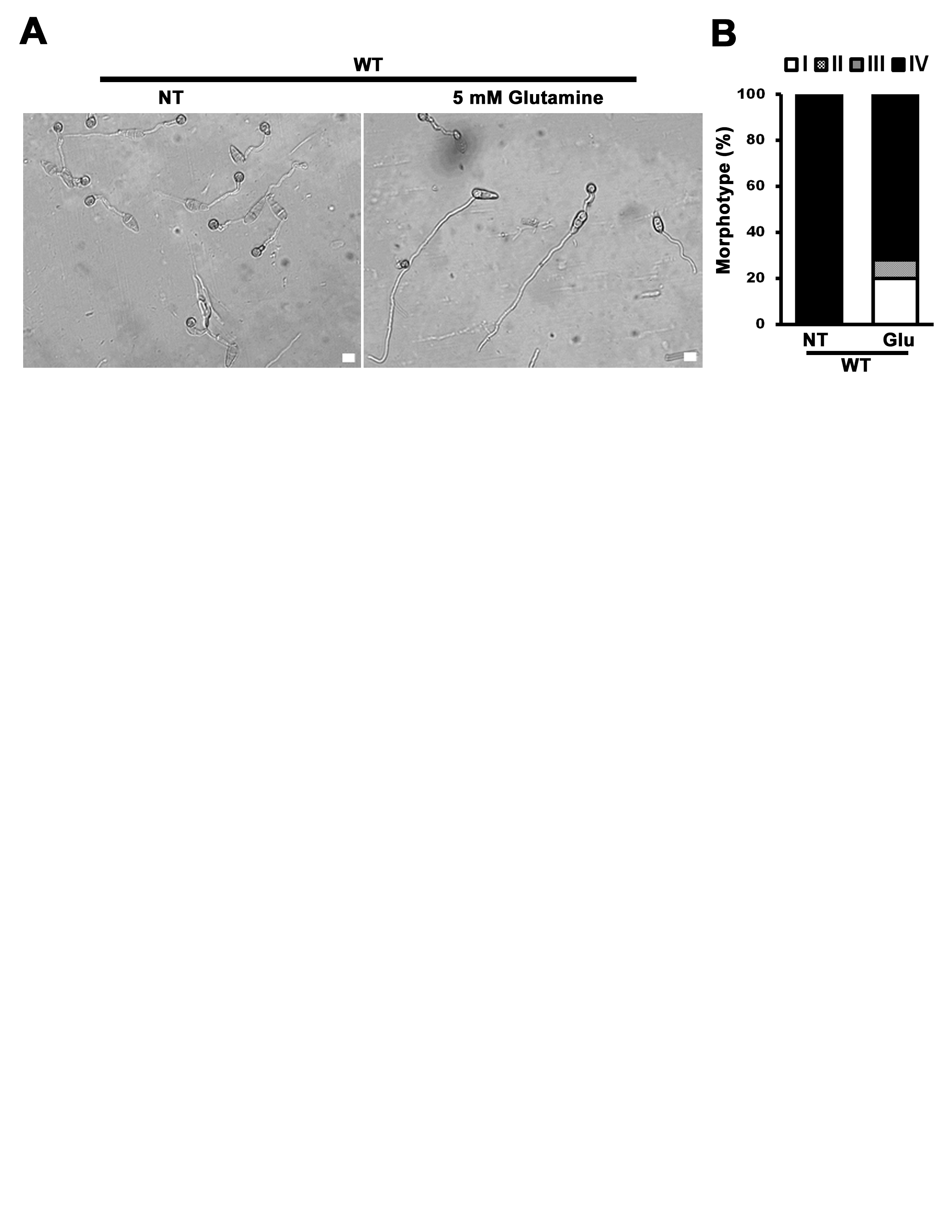

Supplement: S9 Fig — (A) Germinating spores of WT treated, or not treated (NT), with 5 mM glutamine were viewed at 24 hpi. Treatment induced long germ tubes and prevented appressorium formation by some germinating spores. Scale bars are 10 μm. (B) By 24 hpi, compared to NT controls, glutamine treated WT spores displayed the Type I, II and IV morphotypes observed for germinating Δabl1 and glucose treated WT spores (Fig 6C), but not in the same proportions, and absent Type III, suggesting glutamine and glucose might activate TOR by different pathways. The large proportion of treated germinating spores displaying Type IV morphotype by 24 hpi might reflect reduced uptake of glutamine due to its poor solubility in water. Type I-IV morphotypes are designated according to Fig 1C. Gln = 5 mM glutamine. NT = no treatment. (JPG) [file pgen.1006557.s009.jpg]

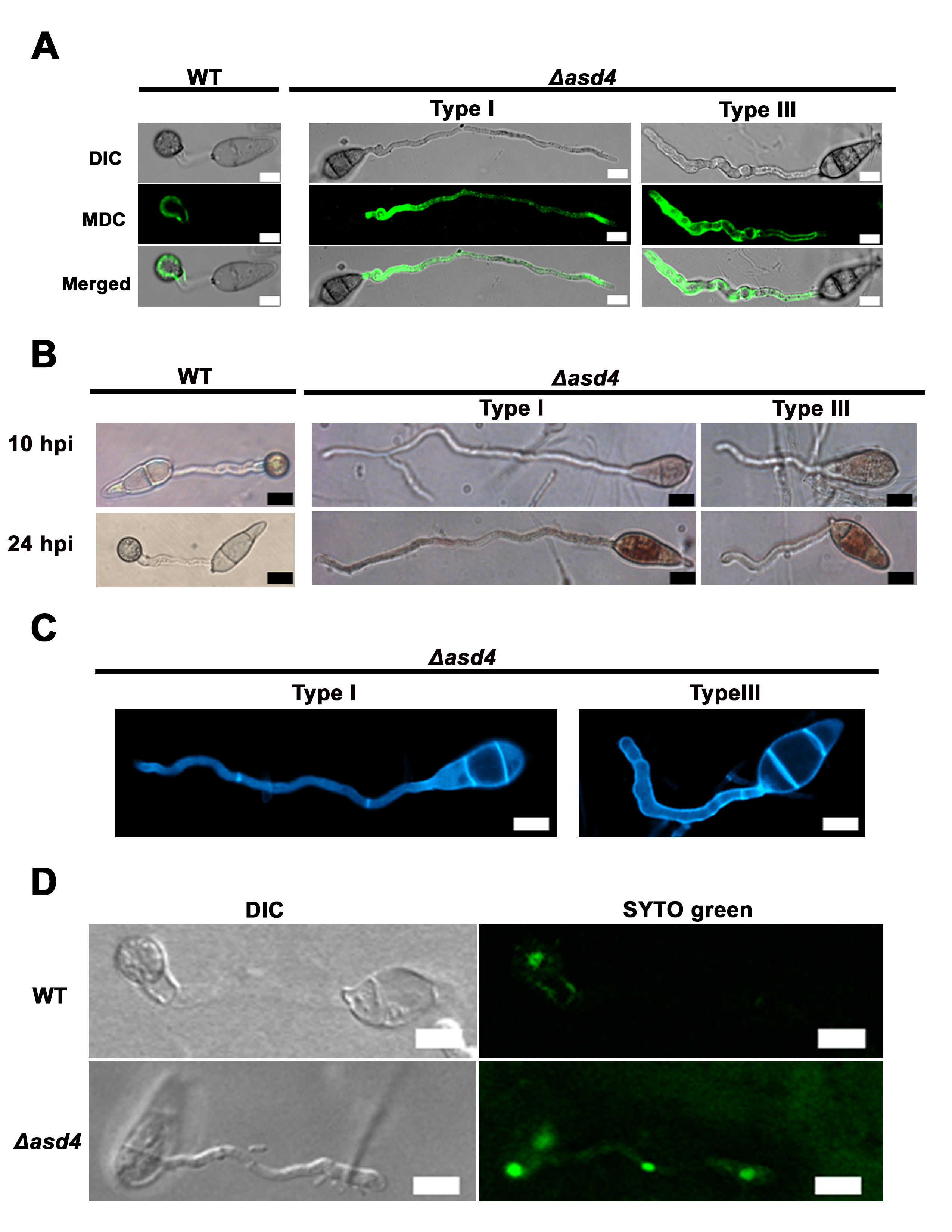

Supplement: S10 Fig — (A) Monodansylcadaverine (MDC) staining shows autophagosome distribution was affected in Δasd4 germinating spores compared to WT by 24 hpi. Type I and III morphotypes were observed for Δasd4 mutants and the autophagosome distribution displayed the same patterns as those of Δabl1. Scale bars are 10 μm. (B) Glycogen was stained by KI and I2 mixture for five minutes at the indicated time points. Arrows indicate the aggregation of glycogen in both Δasd4 and WT germinating spores. By 10 hpi, glycogen had mobilized from WT spores into incipient appressoria, where it was fully degraded by 24 hpi. In contrast, glycogen was still present in the conidial area in both morphotypes of Δasd4 spores by10 hpi and 24 hpi. Stars indicate glycogen accumulation. (C) Δasd4 germinating spores presenting as Type I and III morphotypes displayed more septation events in germ tubes than WT (Fig 6B). Cell walls were stained by calcofluor white and 10% KOH at 24 hpi. Scale bars are 10 μm. (D) Nuclei were visualized by the SYTO green fluorescent nucleic acid stain. 200 μl spore suspension at 1X104 spores/ml, was inoculated onto hydrophobic plastic coverslip and incubated in dark for 24 hours at room temperature. 10 μl of 1:1000 SYTO green (Fisher, USA) solution was added to the spore suspension and gently mixed on the plastic coverslip. Images were taken 30 mins after SYTO green staining by confocal microscopy. The samples were excited with the 488 nm filter and viewed with the 525 nm laser. Scale bar is 10 μm. (JPG) [file pgen.1006557.s010.jpg]
