# Supplementary material for: Glucose-ABL1-TOR Signaling Modulates Cell Cycle Tuning to Control Terminal Appressorial Cell Differentiation
Source: PLoS Genet. 2017 Jan 10;13(1):e1006557. doi: 10.1371/journal.pgen.1006557 (PMC5266329; doi:10.1371/journal.pgen.1006557)
Supplement: S1 Table — (DOCX) [file pgen.1006557.s011.docx]

**S1 Table**. Morphotypes formed by germinating conidia at 24 hours post inoculation on hydrophobic surfaces.

| Strain | Morphotypes | Treatments | | | | | | | |
| --- | --- | --- | --- | --- | --- | --- | --- | --- | --- |
|  |  | NT^a^ | | 100 nM Rap^b^ | | 10 mM cAMP^c^ | | 1% Glc^d^ | |
|  |  | Mean^e^ (%) | S.D.^f^ | Mean^e^  (%) | S.D.^f^ | Mean^e^  (%) | S.D.^f^ | Mean^e^  (%) | S.D.^f^ |
| WT^g^ | I | - | - | - | - | - | - | 22.7 | 3.1 |
|  | II | - | - | - | - | - | - | 4.7 | 2.3 |
|  | III | - | - | - | - | - | - | 47.3 | 3.1 |
|  | IV | 100.0 | 0.0 | 100.0 | 0.0 | 100.0 | 0.0 | 25.3 | 6.1 |
| WT^g^  H1:RFP | I | - | - | - | - | - | - | 11.3 | 3.1 |
|  | II | - | - | - | - | - | - | 4.7 | 2.3 |
|  | III | - | - | - | - | - | - | 46.0 | 8.0 |
|  | IV | 100.0 | 0.0 | 100.0 | 0.0 | 100.0 | 0.0 | 38.0 | 12.5 |
| ∆*abl1* | I | 33.3 | 3.1 | 6.7 | 2.3 | 39.3 | 3.1 | 37.3 | 1.6 |
|  | II | 9.3 | 3.1 | 6.0 | 3.5 | 17.3 | 3.1 | 16.7 | 2.3 |
|  | III | 32.7 | 3.1 | 14.7 | 2.3 | 22.7 | 3.1 | 22.0 | 2.0 |
|  | IV | 24.7 | 5.1 | 72.7 | 3.1 | 20.7 | 6.4 | 24.0 | 2.0 |
| ∆*abl1*  H1:RFP | I | 28.67 | 2.3 | 7.3 | 4.2 | 32.0 | 2.0 | 29.3 | 3.1 |
|  | II | 14.00 | 2.0 | 6.0 | 4.0 | 16.7 | 2.3 | 14.7 | 3.1 |
|  | III | 30.67 | 4.2 | 14.7 | 1.2 | 24.7 | 4.2 | 34.7 | 3.1 |
|  | IV | 26.67 | 8.1 | 72.0 | 5.3 | 26.7 | 5.3 | 21.3 | 4.6 |

^a^ NT: not treated

^b^Rap: rapamycin

^c^cAMP: cyclic AMP

^d^1 % Glc: 1 % (w/v) glucose

^e^ Values correspond to the average of six independent repetitions.

^f^ Standard deviation

^d^ Only Type IV morphotypes were observed in WT strains. Conidia without appressoria did not germinate and were not counted here.
