# Supplementary material for: Glucose-ABL1-TOR Signaling Modulates Cell Cycle Tuning to Control Terminal Appressorial Cell Differentiation
Source: PLoS Genet. 2017 Jan 10;13(1):e1006557. doi: 10.1371/journal.pgen.1006557 (PMC5266329; doi:10.1371/journal.pgen.1006557)
Supplement: S2 Table — (DOCX) [file pgen.1006557.s012.docx]

**S2 Table**. Number of nuclei carried by germinating conidia at 24 hours post inoculation on artificial hydrophobic surfaces at 22 ^o^C.

| Treatment | WT | | | | *∆abl1* | | | |
| --- | --- | --- | --- | --- | --- | --- | --- | --- |
|  | treatment added at (h)^a^ | | | | treatment added at (h)^a^ | | | |
|  | - | 0 | 4 | 8 | - | 0 | 4 | 8 |
| NT^b^ | 1.3 ± 0.24 | - | - | - | 4.3 ± 1.22 | - | - | - |
| CHX^c^ | - | 2.8 ± 0.01 | 2.7 ± 0.01 | 1.3 ± 0.13 | - | 2.8 ± 0.03 | 3.7 ± 0.03 | 4.1 ± 0.12 |
| HU^d^ | - | 2.2 ± 0.23 | 3.2 ± 0.12 | 1.3 ± 0.14 | - | 2.8 ± 0.12 | 2.5 ± 0.01 | ^g^1.6 ± 0.01 |
| BEN^e^ | - | 0.8 ± 0.02 | 1.0 ± 0.03 | 1.4 ± 0.06 | - | 0.6 ± 0.04 | 0.7 ± 0.02 | ^h^1.8 ± 0.01 |
| RAP^f^ | - | 1.1 ± 0.14 | 1.1 ± 0.12 | 1.0 ± 0.12 | - | 1.2 ± 0.02 | 1.4 ± 0.03 | 3.2 ± 0.02 |

^a^ Hours post inoculation at which treatments were added to the spore suspension incubated on the hydrophobic surface. Values correspond to the average of 30 spores per hydrophobic coverslip, repeated in triplicate ± standard deviation. ^b^ NT: No treatment. ^c^ Cyclohexamide [2 mM]. ^d^ Hydroxyurea [50 mM]. ^e^ Benomyl [30 µM]. ^f^ Rapamycin [100 nm]. ^g^ Note that long-term HU exposure promotes some nuclear degeneration in Δ*abl1* strains even when added at later time points. ^h^ Note that Ben treatment induces some nuclear degeneration at all time points.
