# Supplementary material for: Glucose-ABL1-TOR Signaling Modulates Cell Cycle Tuning to Control Terminal Appressorial Cell Differentiation
Source: PLoS Genet. 2017 Jan 10;13(1):e1006557. doi: 10.1371/journal.pgen.1006557 (PMC5266329; doi:10.1371/journal.pgen.1006557)
Supplement: S3 Table — (DOCX) [file pgen.1006557.s013.docx]

**S3 Table**. Percentage of appressoria formed at 24 hpi by germinating WT and *∆abl1* spores at 22 ^o^C.

| Treatment | WT | | | | *∆abl1* | | | |
| --- | --- | --- | --- | --- | --- | --- | --- | --- |
|  | treatment added at (h)^a^ | | | | treatment added at (h)^a^ | | | |
|  | - | 0 | 4 | 8 | - | 0 | 4 | 8 |
| NT^b^ | 87.0 ± 3.60 | - | - | - | 34.0 ± 2.60 | - | - | - |
| CHX^c^ | - | 0.0 ± 0.00 | 1.3 ± 0.63 | 85.3 ± 2.12 | - | 0.0 ± 0.00 | 0.0 ± 0.00 | 4.3 ± 0.64 |
| HU^d^ | - | 1.7 ± 1.52 | 2.3 ± 1.50 | 80.7 ± 1.50 | - | 0.7 ± 0.63 | 5.3 ± 0.622 | 21.7 ±1.50 |
| BEN^e^ | - | 70.3 ± 1.50 | 80.0 ± 2.00 | 81.3 ± 1.50 | - | 80.7 ± 4.22 | 80.0 ± 4.43 | 33.0 ± 1.00 |
| RAP^f^ | - | 82.7 ± 1.50 | 88.7 ± 2.14 | 87.7 ± 3.13 | - | 72.0 ± 1.00 | 67.3 ± 2.12 | 35.0 ± 2.00 |

^a^ Hours post inoculation at which treatments were added to the spore suspension incubated on the hydrophobic surface. Values correspond to the average of 30 spores per hydrophobic coverslip, repeated in triplicate ± standard deviation. ^b^ NT: No treatment. ^c^ Cyclohexamide [2 mM]. ^d^ Hydroxyurea [50 mM]. ^e^Benomyl [30 µM]. ^f^ Rapamycin [100 nm].
