# Supplementary material for: Glucose-ABL1-TOR Signaling Modulates Cell Cycle Tuning to Control Terminal Appressorial Cell Differentiation
Source: PLoS Genet. 2017 Jan 10;13(1):e1006557. doi: 10.1371/journal.pgen.1006557 (PMC5266329; doi:10.1371/journal.pgen.1006557)
Supplement: S4 Table — (DOCX) [file pgen.1006557.s014.docx]

**S4 Table.** *Magnaporthe oryzae* strains used in this study.

| **Strains** | **Genotype** | **Reference** |
| --- | --- | --- |
| Guy11 | *M. oryzae* wild type isolate (WT) used throughout this study | Wilson et al. 2010 |
| Δ*abl1* | WT carrying a deletion of the gene encoding AMPKβ-like protein (MGG_00987) | *This study* |
| Δ*abl1 ABL1* | Complementation strain resulting from integration of the full length *ABL1* gene and native promoter into the genome of strains carrying the Δ*abl1* gene deletion | *This study* |
| Δ*abl1 ABL1^GFP^* | Complementation strain expressing the Abl1^GFP^ fusion protein under its native promoter in the Δ*abl1* mutant background. | *This study* |
| WT H1:RFP | WT expressing a histone H1 fused to a td tomato variant of red fluorescent protein | Saunders et al. 2010a |
| Δ*abl1* H1:RFP | WT H1:RFP strain carrying a deletion of the gene encoding AMPKβ-like protein (MGG_00987) | *This study* |
| Δ*tps1* | WT carrying a deletion of the gene encoding trehalose-6-phosphate synthase 1 (MGG_03860) | Foster et al. 2004 |
| Δ*tps1* Δ*abl1* | Deletion of *ABL1* in the Δ*tps1* mutant background. | *This study* |
| Δ*asd4* | GATA factor (MGG_06050) deletion mutant of Guy11. | Marroquin-Guzman and Wilson, 2015 |
| Δ*rbp35* | WT carrying a deletion of the gene encoding an RNA-binding protein (MGG_02741) | Franceschetti et al. 2011 |
| Δ*abl1* Δ*rbp35* | Deletion of *ABL1* in the Δ*rbp35* mutant background | *This study* |
| Δ*fpr1* | WT carrying a deletion of the gene encoding FKBP12 (MGG_06035) | Marroquin-Guzman and Wilson, 2015 |

Foster AJ, Jenkinson JM, Talbot NJ. 2003. Trehalose synthesis and metabolism are required at different stages of plant infection by *Magnaporthe grisea*. *EMBO J* **22**:225-235.

Franceschetti M, Bueno E, Wilson RA, Tucker SL, Gomez-Mena C, Calder G, Sesma A. 2011. Fungal virulence and development is regulated by alternative pre-mRNA 30 end processing in *Magnaporthe oryzae*. *PLoS Pathog* **7**:e1002441.

Marroquin-Guzman M, Wilson RA. (2015). GATA-dependent glutaminolysis drives appressorium formation in *Magnaporthe oryzae* by suppressing TOR inhibition of cAMP/PKA signaling. *PLoS Pathog* **11**: e1004851.

Saunders DGO, Aves SJ, Talbot NJ. 2010a. Cell cycle-mediated regulation of plant infection by the rice blast fungus Magnaporthe oryzae. *Plant Cell* **22**: 497-507.

Wilson RA, Gibson RP, Quispe CF, Littlechild JA, Talbot NJ. 2010. An NADPH-dependent genetic switch regulates plant infection by the rice blast fungus. *Proc Natl Acad Sci USA* **107**: 21902-21907. doi: 10.1073/pnas.1006839107.
