# Supplementary material for: Glucose-ABL1-TOR Signaling Modulates Cell Cycle Tuning to Control Terminal Appressorial Cell Differentiation
Source: PLoS Genet. 2017 Jan 10;13(1):e1006557. doi: 10.1371/journal.pgen.1006557 (PMC5266329; doi:10.1371/journal.pgen.1006557)
Supplement: S5 Table — (DOCX) [file pgen.1006557.s015.docx]

**S5 Table.** Oligonucleotide primers used in this study.

| **Gene** | **Primer** | **Sequence 5’ – 3’** |
| --- | --- | --- |
| *ILV1^1^* | M13F:IL^2^ | **CGCCAGGGGTTTTCCCAGTCACGAC**GTCGACGTGCCAACGCCACAG |
|  | ILSplit | AAGCATGTGCAGTGCCTTC |
|  | M13R:LV1^2^ | **AGCGGATAACAATTTCACACAGGA**GTCGACGTGAGAGCATGCTAA |
|  | LV1Split | CGCCCGGCCGACATCC |
| *ABL1* | ABL1-LF5’ ^3^ | CCATTAGCGCATGCCCTAC |
|  | ABL1-LF3’ ^2,3^ | **GTCGTGACTGGGAAAACCCTGGCG**GATACAACGGGTTTGGTAAAG |
|  | ABL1-RF5’^2,3^ | **TCCTGTGTGAAATTGTTATCCGCT**TTATTACAAGGTTAGCTCCGATTTG |
|  | ABL1-RF3’ ^3^ | CTTTCGGAGCAGCCTGGTT |
|  | ABL1-nesF ^3^ | CTCTGACGCATACTGACAAGGC |
|  | ABL1-nesR ^3^ | CCACTGGCCGTCGACAAC |
| *ABL1* | QRT-PCR abl1 F^4^ | TCGTCAAGGAGCCTGAACCCA |
|  | QRT-PCR abl1 R^4^ | TGCTCTTGACCTTGCTAATGATGGT |
| *TUB1* | QRT-PCR b-tub F2 ^4^ | CGCGGCCTCAAGATGTCGT |
|  | QRT-PCR b-tub R2 ^4^ | GCCTCCTCCTCGTACTCCTCTTCC |
| *RS2* | qRS2F ^4^ | GGTTGCCTCGCCCGCTG |
|  | qRS2R ^4^ | CGCTTGCCGTCCCTGAGG |
| *RS3* | qRS3F ^4^ | TCGTTCAGCCCGTCAGCCAA |
|  | qRS3R ^4^ | CTCCTGCTCCTCACCCTCACCC |
| *TPS1* | qTPS1-F1 | AGCAGCGCGAGGCCAACTT |
|  | qTPS1-R1 | TCAGTTTCCCTCCGTCTTGTTGTC |
| *ABL1* | ABL1-GF ^5, 7, 8^ | **TATAGGGCGAATTGGGTACTCAAATTGGTT**TGACCGACAGGGACGAGAAGAATA |
|  | ABL1-GR ^5^ | **CCCGGTGAACAGCTCCTCGCCCTTGCTCA**CCGGTTCTTCTTCTTCTTCTCGGCT |
|  | ABL1-nesGF ^6^ | ACGACATGAAACGGTGTGGCAG |
|  | ABL1-nesGR ^6^ | TTGCTCTTGACCTTGCTAATGATGG |

^1^Sulphonylurea resistance gene amplification. ^2^M13F/M13R sequences, highlighted in bold, are upstream of the gene specific sequences, respectively (Wilson et al. 2010). ^3^Primers for split marker deletion construct (Wilson et al. 2010). ^4^Primers used for qRT-PCR analysis. ^5^Nucleotide sequences of Native-C-FP and GFP-C-RP, highlighted in bold, added to the 5’-end of the promoter region and 3’end of the open reading frame of the target gene, respectively (Zhou et al. 2011). ^6^ Primers used to screen yeast colonies carrying plasmids expressing *ABL1*.

Wilson RA, Gibson RP, Quispe CF, Littlechild JA, Talbot NJ. 2010. An NADPH-dependent genetic switch regulates plant infection by the rice blast fungus. *Proc Natl Acad Sci USA* **107**: 21902-21907. doi: 10.1073/pnas.1006839107.

Zhou X, Li G, Xu J-R. 2011. Efficient Approaches for Generating GFP Fusion and Epitope-Tagging Constructs in Filamentous Fungi. Pp. 199-211 in Xu J-R and Bluhm BH (eds.), *Fungal Genomics*: *Methods and Protocols*, Methods in Molecular Biology, vol. **722**, DOI 10.1007/978-1-61779-040-9_15, © Springer Science+Business Media, LLC.
